# Fine-Grained Parcellation of the Macaque Nucleus Accumbens by High-Resolution Diffusion Tensor Tractography

**DOI:** 10.3389/fnins.2019.00709

**Published:** 2019-07-10

**Authors:** Xiaoluan Xia, Lingzhong Fan, Bing Hou, Baogui Zhang, Dan Zhang, Chen Cheng, Hongxia Deng, Yunyun Dong, Xudong Zhao, Haifang Li, Tianzi Jiang

**Affiliations:** ^1^College of Information and Computer, Taiyuan University of Technology, Jinzhong, China; ^2^Brainnetome Center, Institute of Automation, Chinese Academy of Sciences, Beijing, China; ^3^University of Chinese Academy of Sciences, Beijing, China; ^4^National Laboratory of Pattern Recognition, Institute of Automation, Chinese Academy of Sciences, Beijing, China; ^5^Core Facility, Center of Biomedical Analysis, Tsinghua University, Beijing, China; ^6^State Key Laboratory of Brain and Cognitive Sciences, Institute of Biophysics, Chinese Academy of Sciences, Beijing, China; ^7^Center for Excellence in Brain Science and Intelligence Technology, Institute of Automation, Chinese Academy of Sciences, Beijing, China; ^8^The Clinical Hospital of Chengdu Brain Science Institute, MOE Key Lab for Neuroinformation, University of Electronic Science and Technology of China, Chengdu, China; ^9^Queensland Brain Institute, The University of Queensland, Brisbane, QLD, Australia

**Keywords:** neuroimaging, connectivity, ventral striatum, medial shell, lateral shell

## Abstract

Limited in part by the spatial resolution of typical *in vivo* magnetic resonance imaging (MRI) data, recent neuroimaging studies have only identified a connectivity-based shell-core-like partitioning of the nucleus accumbens (Acb) in humans. This has hindered the process of making a more refined description of the Acb using non-invasive neuroimaging technologies and approaches. In this study, high-resolution *ex vivo* macaque brain diffusion MRI data were acquired to investigate the tractography-based parcellation of the Acb. Our results identified a shell-core-like partitioning in macaques that is similar to that in humans as well as an alternative solution that subdivided the Acb into four parcels, the medial shell, the lateral shell, the ventral core, and the dorsal core. Furthermore, we characterized the specific anatomical and functional connectivity profiles of these Acb subregions and generalized their specialized functions to establish a fine-grained macaque Acb brainnetome atlas. This atlas should be helpful in neuroimaging, stereotactic surgery, and comparative neuroimaging studies to reveal the neurophysiological substrates of various diseases and cognitive functions associated with the Acb.

## Introduction

The nucleus accumbens (Acb) is an integral part of the striatal complex (Heimer et al., 1991) and exhibits multi-aspect (e.g., connectivity and function) and multi-level (from macro- to micro-level) heterogeneity (Meredith et al., 1996; Humphries and Prescott, 2010). The various Acb subregions have been recognized as important hubs for integrating different combinations of signals from the prefrontal and limbic areas to serve dissociable roles in refining action selection (Floresco, 2015).

Historically, the boundaries of the Acb were never well-defined histochemically, e.g., the Acb core fades into the ventral caudate and putamen nuclei, especially far up the medial caudate border (Brauer et al., 2000; Neto et al., 2008), but have depended on more modern anatomical analyses for the specifics (see Salgado and Kaplitt, 2015 or Section “Boundaries of the Acb Suggested by Anatomical Analyses” in Supplementary Material). The Acb cannot be directly delineated by magnetic resonance imaging (MRI) due to a lack of distinct signal intensity, but human Acb-like regions have been identified using connectivity-based parcellations of the striatum (Tziortzi et al., 2013; Janssen et al., 2015; Fan et al., 2016). However, these connectivity-defined regions appeared to have significant extensions into the caudate nucleus and putamen compared to the microanatomical-defined Acb and thus are better suited to be considered as a ventral striatum-like or limbic striatum-like region. In short, there is no definitive conclusion about the correspondence between the microanatomical and connectivity boundaries in this region. Thus, we argue that it is necessary to generate a relatively accurate connectivity-based Acb, which, compared to the less well-delineated microanatomically defined Acb, is better suited for being considered as a connection unit (i.e., a collection of voxels having similar connectivity features). Identifying this will enable researchers to perform connection analyses in a reasonable fashion, e.g., tractography-based parcellation (Eickhoff et al., 2015).

Inputs from the prefrontal and limbic areas display differences in topographical organization throughout the Acb (Russchen et al., 1985; Haber et al., 1990; Humphries and Prescott, 2010), leading to speculation that this nucleus consists of separate clusters of cells performing different functional roles, which are determined by their inputs (Pennartz et al., 1994; O’Donnell, 1999). As such, the subdifferentiation of this nucleus has been studied at multiple levels, yielding the well-accepted macroscopic shell-core architecture and mesoscopic patch-matrix organization, as well as many intermediate subdivision solutions (Humphries and Prescott, 2010; Salgado and Kaplitt, 2015). For example, the shell has distinguishable medial and lateral regions (plus a possible intermediate region because of their diffusion boundaries); the medial shell in turn has distinguishable dorsal and ventral parts. Furthermore, the specific inputs and outputs of these Acb subregions serve their specialized functionality and exhibit dissociable roles in refining action selection (Humphries and Prescott, 2010; Floresco, 2015). And thus, these Acb partitions, to various degrees, subserve the anatomical and functional refinements of the limbic-motor interface and the dopamine systems (Ikemoto, 2007; Floresco, 2015; Morrison et al., 2017). However, *in vivo* neuroimaging, partially limited by the spatial resolution of the MRI data, connectivity studies have only identified a shell-core-like partitioning of the Acb in humans (Baliki et al., 2013; Xia et al., 2017; Zhao et al., 2018). Whether such a connectivity-based shell-core-like parcellation exists in non-human animals (e.g., macaques) and whether more detailed connectivity heterogeneity within the Acb, as detected by higher-resolution tractography, can support a finer Acb parcellation than the traditional dichotomization are worth investigating. If it does, fine-grained Acb brainnetome atlases can be established for different species to facilitate more refined descriptions of the Acb and viable cross-species comparisons of these nuclei in future neuroimaging research (Thiebaut de Schotten et al., 2018).

To address these issues, we first acquired high-resolution diffusion MRI data from rhesus macaque brain specimens using 9.4 Tesla (T) scanners. Then, the striatum was recursively parcellated based on probabilistic diffusion tractography to generate a relatively accurate connectivity-based Acb region. Then this connection unit was used as the region of interest (ROI) to detect the parcellation of the macaque Acb and to test the hypothesis that high-resolution tractography can enable a finer subdivision of the Acb than the conventional dichotomy. We further characterized the anatomical and resting-state functional connectivity (rsFC) profiles of these Acb subregions. Their unique connectivity profiles together with earlier research findings were then used to generalize about the possible specialized functionality of these subregions to establish the final fine-grained macaque Acb brainnetome atlas.

## Materials and Methods

### Subjects and MRI Data Acquisition

All experimental procedures were performed in strict accordance with the recommendations in the National Institutes of Health Guide for the Care and Use of Laboratory Animals. All of the animals were handled according to the protocol (#IA-2016-05) approved by the animal care and use committee of the Institute of Automation, Chinese Academy of Sciences (CAS). The *ex vivo* rhesus macaque MRI dataset (MDS1) consisted of 8 adult macaque brain specimens (ages: 4, 4, 5, 6, 8, 12, 15, and 23 years; 2 males; see Section “Preparation of the Specimens and MRI Data Acquisition” in Supplementary Material). These monkeys were obtained from a colony maintained by the Kunming Institute of Zoology (KIZ), CAS and were judged by the institutional veterinarian of KIZ as appropriate subjects for euthanasia due to serious disease. All brain specimens were obtained at necropsy immediately following euthanasia due to reasons not related to the study (see Section “Preparation of the Specimens and MRI Data Acquisition” in Supplementary Material for detailed preparation of the brain specimens). The high-resolution MRI data were acquired on a 9.4T horizontal animal MRI system (Bruker Biospec 94/30 USR; for detailed parameters, Section “MRI Data Acquisition” in Supplementary Material). The diffusion MRI data (TR/TE = 9800/21.8 ms; voxel sizes = 0.6 × 0.6 × 0.6577 mm) included 60 diffusion directions (*b*-value = 1000 s/mm^2^) and 4 non-diffusion gradients acquisition (*b*-value = 0 s/mm^2^). The data quality and availability of these low *b*-value diffusion images in tractography-based parcellation were checked (see Section “MRI Data Quality Checking” in Supplementary Material). We used this dataset to parcellate the striatum and Acb and to characterize the anatomical connectivity profiles of these Acb subregions. The sample size was comparable to those used in earlier parcellation studies (Mars et al., 2012; Wang et al., 2012; Zhuo et al., 2016). In addition, *in vivo* macaque MRI dataset (MDS2; 24 macaques; ages: 3.2–4.4 years; body weight: 5.2–6.887 kg; 20 males) was used in this study to characterize the rsFC profiles of the Acb subregions. Their functional MRI data were acquired on a 3T Siemens Magnetom Verio MR scanner under anesthesia (TR/TE = 2200/3.68 ms; voxel sizes = 1.803 × 1.803 × 1.8 mm; 240 volumes) and have been preprocessed and used in an earlier study (see Wang et al., 2017, for details).

### MRI Data Preprocessing

The preprocessing of the structural images was performed as follows: correction of the distortion due to magnetic field inhomogeneity; non-brain removal for the *in vivo* individuals in MDS2; calculation of the transformations between individual brains and a macaque brain template (Calabrese et al., 2015) using the symmetric normalization transformation model (Avants et al., 2008); and generation of the subject-native tissue maps of the gray matter (GM), white matter (WM), and cerebrospinal fluid (CSF) using the FMRIB Software Library (FSL) FAST program (Zhang et al., 2001) and the prior tissues’ probability maps (Rohlfing et al., 2012).

The distortion caused by eddy current was corrected for the diffusion images using affine registration of all volumes to a target volume with no diffusion weighting. Then the transformations between the structural and diffusion images were calculated using a 6 degrees of freedom (DOF) FSL FLIRT boundary-based registration (BBR; Greve and Fischl, 2009). The brain mask image was brought from structural space to diffusion space to remove the non-brain portions, and the distributions of the diffusion parameters were calculated for each voxel (2 fibers per voxel; Jbabdi et al., 2012). The functional images were analyzed using FSL and custom-made software written in MATLAB. The first 10 volumes were discarded, followed by a slice-timing correction. Motion correction was performed using the MCFLIRT program (Jenkinson et al., 2002). Likewise, the transformations between structural and functional images were calculated using 6 DOF FLIRT BBR, and the non-brain portions were removed. Next, the confounding head movement time series (six motion parameters) and the mean time series of the WM and CSF were regressed out. A 3 mm Gaussian kernel of FWHM was used to spatially smooth the data. Also, the linear and quadratic trends of the fMRI data were removed and independent component analysis-denoising was performed using the MELODIC program (Beckmann and Smith, 2004). Finally, a band-pass filter was used to separate the data at slow-4: 0.027–0.073 Hz (Zuo et al., 2010) to reduce the low-frequency drift and high-frequency noise.

### Defining the Connection Unit of the Macaque Acb

The rhesus macaque striatum excluding the tail was chosen as the seed to perform a tractography-based parcellation. This procedure is similar to the Automatic Tractography-based Parcellation Pipeline program (ATPP RRID: SCR\_014815; Li et al., 2017), and can be described as follows: 1) The seed mask was brought back into the subject-native structural space. After minor manual modifications of the voxels mis-registered into the WM and CSF, this mask was then brought back into individual diffusion space. In subject-native diffusion space, 2) whole-brain probabilistic tractography was implemented for each voxel in the mask using PROBTRACKX2 (Behrens et al., 2007) by sampling 50,000 streamlines to estimate the connectivity probability. Note that the probability counts were corrected by the length of the pathway to compensate for the distance-dependent bias (Tomassini et al., 2007). 3) These path distribution estimates were thresholded at *p* > 0.04% (i.e., 20 out of 50,000 samples) as was done in earlier studies (Fan et al., 2016; Xia et al., 2017; Li et al., 2017) to limit false positive connections and was down-sampled to 1.2 × 1.2 × 1.3154 mm (i.e., we sampled the neighboring four voxels into one by trilinear interpolation) for manageability. 4) All the connectivity probability maps were formed into a connectivity matrix. 5) A cross-correlation matrix between the connectivity profiles of all the voxels in the seed mask was calculated (Johansen-Berg et al., 2004) and was then 6) fed into normalized-cut spectral clustering to subdivide these voxels into multiple subgroups based on the similarity of the connectivity profiles (Baldassano et al., 2015). 7) The voxels in each subgroup were mapped back onto the brain to generate the corresponding subregion. 8) All individual parcellation results were transformed into Montreal Neurological Institute (MNI) monkey space (Frey et al., 2011). In this standard space, for each solution, 9) the most consistent labeling scheme across subjects was adopted to resolve the cluster label mismatch issue caused by the random labeling of the clustering algorithms. Then, 10) groups of locationally corresponding subregions (i.e., those that had the same label) were extracted to generate the probability maps for the subregions. The maximum probability map (MPM) of the seed was calculated by assigning each voxel of the reference space to the area in which it was most likely to be located (Wang et al., 2012).

To avoid spending large amounts of time and resources on the dorsal striatum, which was not the focus of this study, a recursive parcellation procedure was used to parcellate the striatum as follows. In the first parcellation, the cluster number of the spectral clustering algorithm was preset to range from 2 to 8 and the average Cramer’s V (CV) was used to judge the consistency of the spatial distribution of these subregions among individuals as in earlier studies (Fan et al., 2016; Li et al., 2017). The optimal solution was defined by the peak of the average CV, indicating a better split-half reproducibility than the surrounding solutions. Next, a subregion located in the ventral striatum was extracted from the optimal solution for the subsequent recursive parcellations, similar to the procedure described previously (Neubert et al., 2014, 2015), dividing the region into two smaller subdivisions and subsequently further subdividing the resulting areas through many steps. The prior location information of the anatomical Acb was used as a reference to decide which subregion was suited to be chosen as the final connectivity-based Acb.

### The Parcellation of the Macaque Acb

The Acb region extracted from the MPM produced by tractography-based parcellation, but not the microanatomically defined Acb, was used as the seed for the subsequent tractography-based parcellation, to identify the fine subdivisions of the macaque Acb. To take advantage of detailed connectivity heterogeneity within the Acb detected by high resolution tractography, the tractography-based parcellation of the macaque Acb at a relative fine-grained resolution was performed using those original tractographic images have not yet been down-sampled (see Section “Necessary Measures to Parcellate the Acb” in Supplementary Material). We preset the maximum number of clusters to 5 because of the relatively small volume size of this nucleus and its common macroscopic subdivision solutions (2, 3, or 4 clusters) in previous anatomical and histochemical studies. Using the same calculation procedure described above, the optimal solution(s) was determined by the average CV. Finally, the group-level probability map for each Acb subregion was calculated, and the MPM of the Acb was generated in the optimal solution(s). The binary images of the Acb subregions were extracted from the MPM in the optimal solution as ROIs for the subsequent characterization of the Acb subregions.

### Characterization of the Macaque Acb Subregions

The Acb subregions were brought back into subject-native diffusion and function spaces. In subject (MDS1) diffusion space, the whole-brain connectivity probability map (50,000 samples; probability counts were corrected by the length of the pathway) was generated for each Acb subregion and then thresholded to reduce false positive connections. All individual maps and their binary images were transformed into MNI monkey space. We used these individual maps to generate a group-level averaged connectivity probability map and used their binary images to generate a probability fiber tract map. As in previous studies (Fan et al., 2016; Xia et al., 2017), to further reduce false positive connections and the effects of individual differences, the probability fiber tract map was thresholded at *p* > 50% to generate the common fiber tract map. This step is, in effect, analogous to a one-sample *t*-test to determine the voxels that have significant connectivity with the given subregion. We used this common fiber tract map to mask the averaged connectivity probability map.

For each subregion in subject (MDS2) function space, the Pearson correlation coefficients between the mean time series of the given subregion and the time series of each voxel in the GM mask was calculated to generate the rsFC map. Note that the mean time series of these Acb subregions were calculated using fMRI data without smoothing. This map was converted to *z*-values using Fisher’s *z*-transformation and transformed into MNI monkey space. All the normalized *z*-valued rsFC maps were fed into a random effects one-sample *t*-test to determine the regions that had significant correlations with the given subregion. A statistical threshold of *p* < 0.05 (uncorrected) was set to achieve a corrected cluster-wise statistical significance of *p* < 0.05. The cluster size was estimated on the basis of the GM mask and the group-averaged Gaussian filter width. Then, a minimum statistic test for conjunction (Nichols et al., 2005) was performed among these subregions so that the surviving voxels had significant rsFC with all the subregions. The extended threshold for the cluster size of the conjunction was set at 50.

A set of target regions was used to characterize the anatomical and functional connectivity profiles of the Acb subregions. These targets were extracted from a histological rhesus macaque brain atlas (Paxinos et al., 2009), which was transformed into MNI monkey space by Calabrese et al. (2015). In view of the low-resolution MRI data and the unavoidable errors in data acquisition (e.g., noise) and processing (e.g., smoothness and registration errors), this atlas was down-sampled by combining small subdivisions into their parent structures. For instance, hippocampal subregions were combined into a single structure. Then, 17 brain regions having strong connectivities with the Acb subregions (see Section “The Ventral Striatal Subregions” in Supplementary Material or Xia et al., 2017 for detailed criteria) were used to calculate the anatomical and functional connectivity fingerprints of the Acb subregions to represent their connectivity profiles. The final target group included area 10, located in the frontal pole, areas 11 and 13, located respectively, in the mediorostral and mediocaudal parts of the orbitofrontal cortex (OFC), areas 14 and 25 located in the middle and caudal parts, respectively, of the medial prefrontal cortex, area 32 located in the perigenual anterior cingulate cortex, temporal pole (TP), insular cortex (INS), entorhinal cortex (EC), caudate nucleus (Ca), putamen (Pu), pallidum (Pa), hippocampus (Hipp), amygdala (Amyg), hypothalamus (hTha), mediodorsal part of the thalamus (MD), and midbrain (MidB).

We used a set of connectivity ratios to build the connectivity fingerprint and calculated the connectivity ratio as follows: given a target, the connectivity strength (i.e., anatomical connectivity probability or functional coupling) between this target and each Acb subregion was first calculated using the above-mentioned averaged connectivity probability map or significant rsFC map. Then, the connectivity ratio of one of the Acb subregions was defined as:

$$\mathrm{CR}\,\left(\mathrm{target},\mathrm{seed}\,\left(i\right)\right)=\frac{\mathrm{CS}\,\left(\mathrm{target},\mathrm{seed}\,\left(i\right)\right)}{\sum_{j=1}^{n}\mathrm{CS}\,\left(\mathrm{target},\mathrm{seed}\,\left(i\right)\right)}$$

where, seed(*i*) is one of the n Acb subregions; CS(target, seed(*i*)) is the connectivity strength between the given target and seed (*i*); CR(target, seed(*i*)) is the connectivity ratio of seed(*i*).

### Comparisons Across Modalities and Subregions Using Fingerprints

Recent comparative neuroimaging studies have used fingerprints to investigate the relationships between differences in the organization of different regions or brains (Mars et al., 2016). For each Acb subregion, we defined the null hypothesis as “a region’s anatomical and functional connectivity profiles were convergent.” Then, we generated the group-averaged anatomical (8 macaques in MDS1) and functional (24 macaques in MDS2) connectivity fingerprints for this Acb subregion and calculated the observed cosine similarity between the group-averaged anatomical and functional connectivity fingerprints. Subsequently, we performed the following procedure 1000 times to create the permutation distribution: (1) We merged the 8 anatomical and 24 functional connectivity fingerprints and then randomly divided it into two groups (the sample sizes were kept constant, i.e., 8 and 24); (2) We generated the group-averaged fingerprints and calculated their cosine similarity. Finally, test criterion was calculated at the 5% significance level to determine whether the observed cosine similarity was a rare value in the permutation distribution. If the null hypothesis was true, the two groups of the (anatomical and functional) connectivity fingerprints would have the same distribution, and the observed cosine similarity would not be a rare cosine similarity value in the permutation distribution. Using the above permutation test procedure, we also analyzed the similarity of the connectivity fingerprints between the Acb subregions. In addition, for each target in the connectivity fingerprints, paired *t*-tests at the 5% significance level were used to test the significance of the single connectional differences between the results for each pair of the Acb subregions.

## Results

### Connectivity-Based Region of the Acb

**FIGURE 1:**
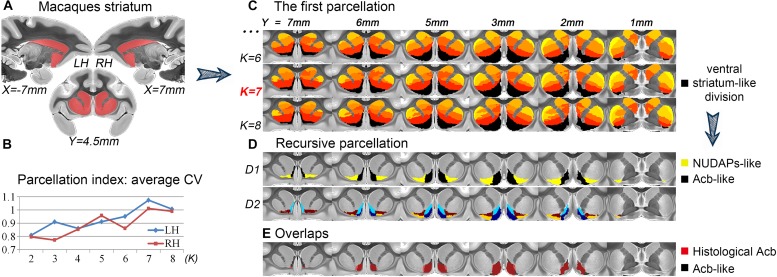
Recursive tractography-based parcellation of the striatum. **(A)** The macaque striatum excluding the tail was chosen as the seed mask. **(B)** In the first parcellation, the parcellation index of the average CV indicated that the 7-cluster (*K* = 7) solution was the optimal data description. **(C)** The resulting MPMs of the first parcellation are shown in a multi-slice presentation. The black cluster in the 7-cluster solution was considered as the ventral striatum-like division. **(D)** The recursive parcellation of the ventral striatum-like division. We first obtained the medial (Acb-like) and lateral (NUDAPs-like) subregions (D1) and then found their dorsoventral subdivisions (D2). **(E)** The high overlap between the resulting Acb-like division and the previous histological Acb region. All the coordinates are shown in MNI monkey space.

After the first tractography-based parcellation of the macaque striatum (Figure 1A), the 7-cluster solution presented the best CV-based data description for both brain hemispheres (Figure 1B) and was thus accepted as the optimal solution. In addition, we found that the ventral cluster (the black cluster in Figure 1C) of the striatum in the 7-cluster solution overlapped considerably with those in the 6- and 8-cluster solutions. Given the above, we considered this cluster to be a stable region from a connectional perspective and extracted this striatal subregion from the MPM in the optimal 7-cluster solution. We named this striatal subregion as the ventral striatum-like division for its good correspondence with the anatomically- described ventral striatum, comprising the Acb, the broad continuity between the caudate nucleus and putamen ventral to the rostral internal capsule, the olfactory tubercle (Heimer et al., 1999), and the medial caudate nucleus from a connectional perspective (Haber and McFarland, 1999).

We further parcellated the ventral striatum-like division to find a more accurate definition of the Acb. In the subsequent recursive parcellation, we found that this cluster could be parcellated into medial and lateral parts. The medial subregion as visualized in the binary image extracted from the MPM presented a high Dice coefficient with the histological Acb (left hemisphere, LH: 84.4%; right hemisphere, RH: 83.6%; see Figure 1E); the lateral subregion, however, presented a low Dice coefficient with the histological Acb (LH: 9%; RH: 7.3%) but corresponded to the histological “neurochemically unique domains of the accumbens and putamen (NUDAPs).” The NUDAPs comprise many patch-like areas located in the ventral border of the Acb and the ventral one-third of the putamen and stand out in their distribution pattern of *u*-opioid, *k*-opioid, and D1-like dopamine receptors (Voorn et al., 1996; see Supplementary Figure S4). Further, the medial and lateral subregions both presented dorsoventral subdivisions, but not further medio-lateral subdivisions (Figure 1D). That means that, after two iterations, only one sagittal-like surface was found to subdivide the ventral striatum-like cluster into medial and lateral subregions. Given this, we considered the medial subregion to be the connectivity-based Acb, and named it the Acb-like division while naming the lateral subregion the NUDAPs-like division.

### Tractography-Based Parcellation of the Acb

**FIGURE 2:**
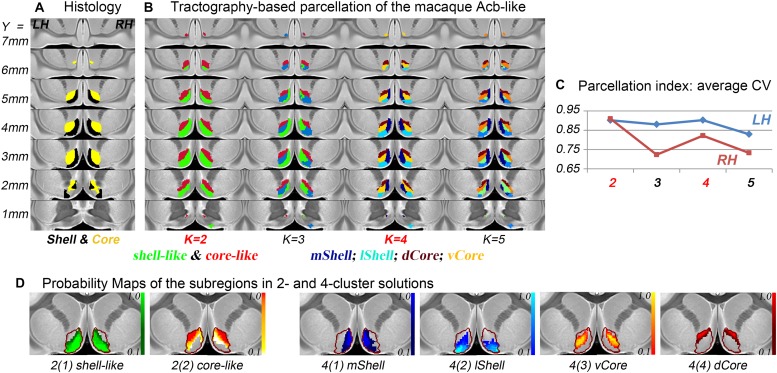
The subdivisions of the macaque Acb. **(A)** The histological shell-core architecture. **(B)** The connectivity-based parcellation results of the Acb-like division in MNI monkey space. **(C)** The parcellation index of the average CV indicates that the 2- and 4-cluster solutions describe the data well. **(D)** The probability maps of the Acb-like subregions: The Acb-like border is indicated by a red curve.

The connection unit of the macaque Acb-like division was further parcellated into 2, 3, 4, and 5 subregions. From the data indices of the average CV, the 2- and 4-cluster solutions, which had high individual consistency, were considered as alternative optimal solutions (Figure 2). Specifically, the Acb-like cluster was first subdivided into ventromedial and dorsolateral parts that showed good correspondence with the histological macaque shell and core, respectively, as well as with the parcellation results in humans (Baliki et al., 2013; Xia et al., 2017; Zhao et al., 2018). The two subregions extracted from the MPM in the 2-cluster solution were thus named the shell-like and core-like divisions. Next, it seemed that the shell-like region was subdivided into a medial and a lateral part, corresponding well to the medial and lateral shell described by Humphries and Prescott (2010), and thus were extracted and named the mShell and lShell divisions, respectively. The core-like region was further subdivided into a dorsal and a ventral part, which were extracted and named the dCore and vCore divisions, respectively. Note that the vCore division may also be seen as a transitional region between the shell-like and core-like divisions. In conclusion, the parcellation results revealed that the macaque Acb has a connectivity-based shell-core-like partitioning that is similar to that in humans. The results also confirmed our hypothesis by providing an alternative optimal 4-cluster solution to the conventional dichotomy.

### Characterization of the Shell-Like and Core-Like Divisions

**FIGURE 3:**
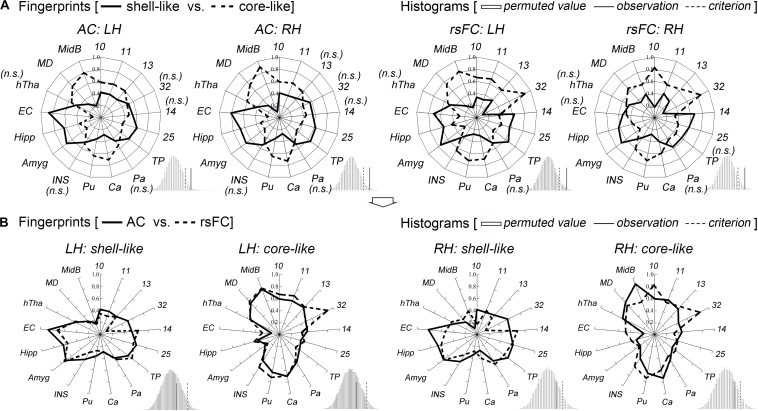
Characterization and comparisons of the two macaque Acb subdivisions. **(A)** The anatomical connectivity (AC) fingerprints of the shell-like and core-like subregions were compared and are shown in the two left panels; the rsFC fingerprints of these two subregions were compared and are shown in the two right panels. For each target area, the connectional differences between the two subregions with this target area were tested using a paired samples *t*-test (AC/rsFC: 8/24 subjects). “n.s.” indicates that no significant difference was found. For the fingerprint, the permutation test (histogram) indicated that the observed value was a rare cosine similarity in the permutation distribution, as can be seen in the right tail of the histogram, the observed cosine similarity was higher than the calculated criterion at the 5% significance level, the null hypothesis was rejected and the two fingerprints were considered to differ. **(B)** For each subregion, the AC and rsFC fingerprints were extracted and compared with each other. The permutation test verified their convergence by indicating that the observed cosine similarity between the two fingerprints was not a rare value in the permutation distribution, as can be seen in the histogram, the observed cosine similarity was lesser than the calculated test criterion at the 5% significance level in the right tail. Thus, we accepted the null hypothesis and indicated that the AC and rsFC fingerprints are “close” to each other. TP, temporal pole; Pa, pallidum; Ca, caudate nucleus; Pu, putamen; INS, insular cortex; Amyg, amygdala; Hipp, hippocampus; EC, entorhinal cortex; hTha, hypothalamus; MD, mediodorsal part of the thalamus; MidB, midbrain.

The shell-like division showed significantly stronger anatomical connectivity probabilities and functional coupling patterns, i.e., rsFC, than the core-like division with areas 14 and 25 and with some limbic structures of the Amyg, Hipp, and EC. In contrast, the core-like region showed significantly stronger connections with areas 10, 11, 13, 32, and the subcortical Ca, Pu, MD, and MidB (Figure 3 and Supplementary Figure S6). These neuroimaging connections are consistent with previous tracing results (Poletti and Creswell, 1977; Van Hoesen et al., 1981; Russchen et al., 1985; Haber et al., 1995; Chikama et al., 1997; Zahm, 1999; Ferry et al., 2000; Stopper and Floresco, 2011; Mavridis and Anagnostopoulou, 2013; Loonen and Ivanova, 2016) and are provided here simply as confirmation. For instance, the cortical input to the shell primarily originates within the medial prefrontal cortex and the medial edge of the OFC (corresponding to areas 14 and 25 in this study), while cortical projections to the core primarily originate within the rest of the OFC and the dorsal prelimbic area (corresponding to areas 11, 13, and 32) (Haber et al., 1995; Ferry et al., 2000). The hippocampal projections from the subiculum and CA1 regions were notably restricted to the shell via the fimbria-fornix fiber bundle (Poletti and Creswell, 1977). The caudal basolateral and rostral basal amygdaloid fibers were found to project throughout the ventral striatum, especially the medial part of the striatum (Russchen et al., 1985).

Shifting attention to the complete connectivity architecture, i.e., the fingerprint, the permutation tests indicated that the observed cosine similarity between the two fingerprints for the shell-like and core-like divisions was greater than the criterion in the right tail (histograms in Figure 3A). Thus, we rejected the null hypothesis that the two fingerprints were “far” from each other. That is, the shell-like and core-like divisions had distinct connectivity profiles and distinct functional connectivity profiles. On the other hand, from the subsequent comparisons between the fingerprints from the two modalities, we concluded that both the shell-like and core-like divisions had convergent (or comparable) anatomical and functional connectivity profiles (Figure 3B).

### Characterization of the mShell, lShell, dCore, and vCore

**FIGURE 4:**
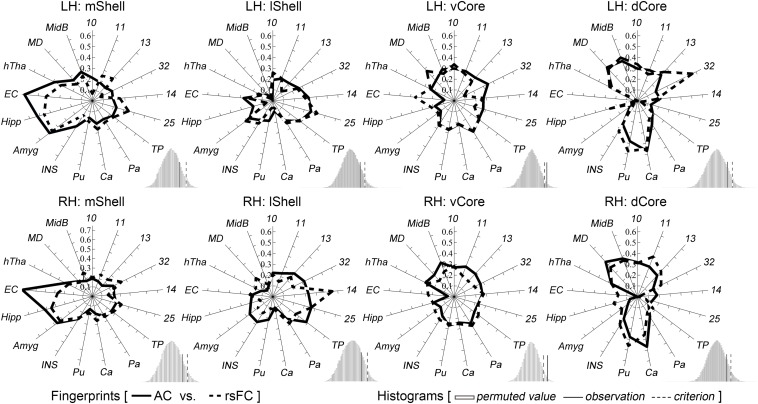
Characterization and comparisons of the four macaque Acb subdivisions. The permutation tests (histograms) indicate that the mShell, lShell, and dCore, but not the vCore, have convergent anatomical and functional connectivity fingerprints. For a detailed legend, please refer to Figure 3.

The above target group was directly used to calculate the anatomical and functional connectivity fingerprints of the four Acb parcels: the mShell, lShell, dCore, and vCore (Figure 4). From a connectivity fingerprint perspective, the mShell and lShell appear to correspond to the subcortical and cortical parts, respectively, of the fingerprint of the dichotomous shell-like; the dCore corresponds to the fingerprint of the dichotomous core-like division; the locational transition region of the vCore, however, seems to also be a connectional transition region because of its relatively uniform connections with almost all these targets. Specifically, we found that the mShell had relatively strong anatomical connectivity and functional coupling with the Amyg, Hipp, and EC; the lShell had relatively strong connections with areas 14, 25 and the TP, whereas the dCore, like the core-like division, had relatively strong connections with areas 10, 11, 13, 32, and the subcortical Ca, Pu, MD, and MidB. Also, some of these unique neuroimaging connectional trends are consistent with previous tracing results (note that some of the experiments were performed on rodents). For instance, the Hipp subiculum and medial EC primarily project to the caudal and rostral parts, respectively, of the medial Acb (corresponding to the mShell, similarly hereinafter) (Groenewegen et al., 1990; Jay and Witter, 1991; Totterdell and Meredith, 1997), whereas only the dorsal subiculum and ventral CA1 regions provided any detectable projections to the rostrolateral shell (rostral lShell) (van Groen and Wyss, 1990; Swanson and Cowan, 1977). The parvicellular division of the Amyg and the medial orbitofrontal cortex project to the medial Acb (mShell), whereas the magnocellular division of the Amyg and the lateral orbitofrontal cortex project to the lateral ventral striatum (lShell, dCore, and vCore) (Russchen et al., 1985). In rats, van Dongen et al. (2005) indicated that the shell-to-core projections were primarily restricted to the border region between the shell and core. This finding supported the unique connectivity-based region of the vCore. In fact, we also found that the other three Acb subregions all presented significantly stronger rsFC with the vCore than any of the other rsFCs between the three Acb subregions (Supplementary Figure S7). Finally, switching back to the connectivity fingerprint, the permutation tests suggested that all of the Acb subregions, except the vCore, had convergent (or comparable) anatomical and functional connectivity profiles.

## Discussion

In this study, high-resolution diffusion tractography was used to make an accurate delineation of the connection unit of the macaque Acb and its fine-grained partitioning. The connectivity-based Acb-like and NUDAPs-like divisions were defined and used for subsequent connectivity analysis. The high-resolution diffusion images enabled us to adequately identify the macroscopic connectional heterogeneity within the shell and core, which helped us to establish the fine-grained macaque Acb atlas by subdividing this nucleus into 2 or 4 subregions. We characterized the unique connectivity profiles of these Acb subregions and analyzed their possible functions to establish a macaque Acb brainnetome atlas.

### Considerations of the Methodological Basis

Local microstructural features (e.g., cyto-, myelo-, and chemo-architecture) and the holistic connective architecture are indicated to be complementary and correlative (cf. Barbas and Rempel-Clower, 1997; van den Heuvel et al., 2015; Cerliani et al., 2016), which are both implicated in determining a region’s function. However, the gross correspondence between these features still remains unclear; different delineation characteristics may result in inconsistent spatial boundaries of brain regions or even different partitioning scheme, as shown by the different parcellations of the auditory cortex using cyto-, myelo-, and chemo-architecture (Moerel et al., 2014) and of the inferior parietal lobule using cytoarchitecture, receptor distribution, and tractography (Caspers et al., 2006, 2013; Mars et al., 2012; Wang et al., 2012). Thus, the histochemically and anatomically defined brain regions (e.g., the Acb and its subregions) may not be connection units, and thus may not be the optimal selection for the regions of interest for connection analyses. But these microstructurally defined heterogeneous information of brain regions in previous invasive animal experiments can offer predictions for similar connectivity-based subdivisions in the human. In addition, many researchers believe that the functions of brain regions depend more on their connectivity architecture than on their microanatomical features (Mesulam, 2006; Knosche and Tittgemeyer, 2011; Cloutman and Lambon Ralph, 2012; Glickfeld et al., 2013). For these reasons, it is necessary to delineate the connection units of the ventral striatal components, e.g., the Acb, to deal with the ever-increasing connection analyses in a more rational way. Therefore, macroscopic tractography, which has been confirmed by previous studies to identify a connectional region (Cohen et al., 2009; Neubert et al., 2014; Wang et al., 2015), was used to delineate the connectivity-based Acb atlas.

### Connectivity-Defined Striatal Components

A recursive parcellation procedure, proposed and validated by previous studies (Beckmann et al., 2009; Mars et al., 2013; Neubert et al., 2014), was used to parcellate the striatum. The macaque ventral striatum-like division, together with the corresponding clusters from previous parcellations of the human striatum (Tziortzi et al., 2013; Janssen et al., 2015; Fan et al., 2016), suggest the uniqueness of the ventral striatum from the perspective of macroscopic connectivity architecture. But unlike previous studies, our goal was to reliably define the connection unit of the Acb by subdividing this ventral striatum-like division. The high amount of overlap between the connectional and histological Acb, together with the identifiability of the histological NUDAPs by their connectivity architecture, may be considered to be additional evidence of the correlation between macroscopic connectivity and microanatomical features (Scannell et al., 1995; Barbas and Rempel-Clower, 1997; Passingham et al., 2002; Hilgetag and Grant, 2010). In addition, we believe that the connection units of the striatal components identified in this study will be useful for node definitions in future neuroimaging connection analyses.

In an earlier study (Xia et al., 2017) using high-quality MRI data provided by the Human Connectome Project (Van Essen et al., 2013), we failed to identify a finer parcellation of the human Acb beyond the known shell-core dichotomy. We attributed this failure in part to the low discriminatory potential of low-resolution diffusion tractography, which was unable to detect subtle connectivity differences within the Acb shell and core. High-resolution *ex vivo* macaque diffusion images were chosen to retest it. Gratifyingly, this dataset confirmed our hypothesis by providing the 4-cluster solution as a second optimal solution along with the 2-cluster one. The shell-like and core-like divisions in the 2-cluster solution have similar morphological distributions and connectivity profiles to those of found in previous anatomical and histochemical studies (Humphries and Prescott, 2010; Salgado and Kaplitt, 2015) and were also similar to those derived from human neuroimaging results (Baliki et al., 2013; Xia et al., 2017; Zhao et al., 2018). Therefore, the specific functions of the two human Acb subregions, summarized in our earlier study (Xia et al., 2017) by their unique neuroimaging connections, together with previous lesion or inactivity experiments in animal models can, to a great extent, be mapped into the corresponding macaque Acb subregions. Specifically, we contend that the macaque shell-like division, having strong connections with area 25, the Amyg, Hipp, and EC, is functionally devoted to aiding the organism in attaining motivationally relevant goals by suppressing lesser- or non-reward stimuli (Floresco, 2015). In contrast, the macaque core-like division, having strong connections with areas 10, 11, 13, the THA, and MidB, appears to be functionally involved in switching from the current stimulus to a more rewarding one, after a subjective Pavlovian prediction about stimuli or cues in a complex environment (Floresco, 2015). In short, the distinctive connectivity of the two Acb subregions contributes to their respective functions by refining action selection in a dissociable manner.

### Fine-Grained Parcellation of the Acb

The shell has distinctive medial and lateral parts and maybe an intermediate part, because of its gradually changing characteristics in primates (Meredith et al., 1996) and rats (Herkenham et al., 1984; Jongen-Rêlo et al., 1994; Ikemoto et al., 2005; Ikemoto, 2007; van der Plasse et al., 2012). Our results indicated that a similar mediolateral distinction in the Acb (i.e., the mShell and lShell, as well as the NUDAPs-like divisions) can be made on the basis of the macroscopic whole-brain connectivity architecture, as well as on the basis of the projectional, histological, morphological, and functional characteristics mentioned in the above studies. In contrast, the core has had inconsistent reports of subdivisions such as various reports of rostrocaudal, mediolateral, and patch-matrix areas (Humphries and Prescott, 2010). We provided a new dorsoventral division of this region using connectivity architecture and showed that the vCore may also be seen as a connectional transition region between the shell and core. Next, in the same way as described above, we summarized the possible specialized functionality of these Acb subregions on the basis of their unique anatomical and functional connectivity together with the results from previous lesion or inactivity experiments involving these regions in animal models.

### Putative Functions of the Fine-Grained Acb Subregions

The convergent projections from the prefrontal cortex and the limbic structures to the medial shell (i.e., mShell) are believed to possess multiple functional roles, e.g., in switching the global mode of behavior away from free-feeding when necessary and in computing the value of stimulus-outcome pairs (Yin and Knowlton, 2006; Ikemoto, 2007; Humphries and Prescott, 2010). In addition, the large amount of concentrated Hipp and EC inputs carrying spatial location information to the mShell suggest that a large number of lesser- or non-rewards processed at these locations may be suppressed in this region (Floresco, 2015; Sjulson et al., 2018). The lShell has few prominent neuroimaging connections, and its counterpart in rats was found to project only to the MidB (lateral VTA and substantia nigra pars compacta) and ventrolateral part of the ventral Pa in tracing studies (Zhou et al., 2003; Ikemoto, 2007). Functionally, this region receives information from the prefrontal cortex, magnocellular Amyg, and, to a small degree, the Hipp about outcomes of previous and current actions (Humphries and Prescott, 2010; Floresco, 2015) and may thus be involved in the complex function of comprehending current stimuli and predicting their appetitive or aversive consequences to adjust the motivation level (Cardinal and Everitt, 2004). As mentioned above, the counterpart of the vCore in rats was defined by its intra-accumbens projection patterns (van Dongen et al., 2005). This region was considered to be both a locational and connectional transition region and thus, functionally, may be involved in the re-integration of information. In addition, an area in the approximate location of the vCore was suggested to be functionally involved in the selection of direction, modulated by the estimated likelihood of reaching the spatial goal from the current position, while another area in the approximate location of the dCore was suggested to be involved in the selection of direction, as modulated by the value of previous outcomes from choosing that direction from the current position (Humphries and Prescott, 2010). In neuroimaging, the dCore inherits the unique connectivity patterns of the core-like division. Thus, the function of the core-like region, inferred by its unique connections, was also maintained by the dCore cluster as well. We also believed that the dCore cluster may be a better target site or node than the core-like cluster in core-related research.

## Conclusion

A tractography-based recursive parcellation of the striatum was performed in macaques to delineate a relatively accurate connectivity-based Acb region. Using this region, we identified connectivity-based shell-core-like partitioning in macaques that is similar to that in humans and verified the hypothesis that high-resolution tractography enables the identification of finer Acb subdivisions, beyond the well-documented dichotomous shell-core architecture. We further characterized the unique anatomical and functional connectivity profiles of these Acb subregions, generated their possible specialized functionalities, and finally established a fine-grained macaque Acb brainnetome atlas.

## Data Availability

All datasets generated for this study are included in the manuscript and/or the Supplementary Files.

## Ethics Statement

All experimental procedures were performed in strict accordance with the recommendations in the National Institutes of Health Guide for the Care and Use of Laboratory Animals. All of the animals were handled according to the protocol (#IA-2016-05) approved by the animal care and use committee of the Institute of Automation, Chinese Academy of Sciences.

## Author Contributions

TJ and HL conceived and designed the study. BH, BZ, DZ, and XZ acquired and checked the data. XX, LF, CC, HD, and YD performed research and analyzed the data. XX, LF, HL, and TJ wrote the manuscript. All authors reviewed and approved the submitted manuscript.

## Conflict of Interest Statement

The authors declare that the research was conducted in the absence of any commercial or financial relationships that could be construed as a potential conflict of interest.

## References

[B1] Avants B. B. Epstein C. L. Grossman M. Gee J. C. (2008). Symmetric diffeomorphic image registration with cross-correlation: evaluating automated labeling of elderly and neurodegenerative brain. *Med. Image Anal.* 12 26–41. 10.1016/j.media.2007.06.004 17659998 PMC2276735

[B2] Baldassano C. Beck D. M. Fei-Fei L. (2015). Parcellating connectivity in spatial maps. *PeerJ.* 3:e784. 10.7717/peerj.784 25737822 PMC4338796

[B3] Baliki M. N. Mansour A. Baria A. T. Huang L. Berger S. E. Fields H. L. (2013). Parceling human accumbens into putative core and shell dissociates encoding of values for reward and pain. *J. Neurosci.* 33 16383–16393. 10.1523/JNEUROSCI.1731-13.2013 24107968 PMC3792469

[B4] Barbas H. Rempel-Clower N. (1997). Cortical structure predicts the pattern of corticocortical connections. *Cereb. Cortex* 7 635–646. 10.1093/cercor/7.7.635 9373019

[B5] Beckmann C. F. Smith S. M. (2004). Probabilistic independent component analysis for functional magnetic resonance imaging. *IEEE. Trans. Med. Imaging* 23 137–152. 10.1109/TMI.2003.822821 14964560

[B6] Beckmann M. Johansen-Berg H. Rushworth M. F. (2009). Connectivity-based parcellation of human cingulate cortex and its relation to functional specialization. *J. Neurosci.* 29 1175–1190. 10.1523/JNEUROSCI.3328-08.2009 19176826 PMC6665147

[B7] Behrens T. E. Berg H. J. Jbabdi S. Rushworth M. F. Woolrich M. W. (2007). Probabilistic diffusion tractography with multiple fibre orientations: what can we gain? *Neuroimage* 34 144–155. 10.1016/j.neuroimage.2006.09.018 17070705 PMC7116582

[B8] Brauer K. Hausser M. Hartig W. Arendt T. (2000). The core-shell dichotomy of nucleus accumbens in the rhesus monkey as revealed by double-immunofluorescence and morphology of cholinergic interneurons. *Brain Res.* 858 151–162. 10.1016/S0006-8993(00)01938-7 10700608

[B9] Calabrese E. Badea A. Coe C. L. Lubach G. R. Shi Y. Styner M. A. (2015). A diffusion tensor MRI atlas of the postmortem rhesus macaque brain. *Neuroimage* 117 408–416. 10.1016/j.neuroimage.2015.05.072 26037056 PMC4512905

[B10] Cardinal R. N. Everitt B. J. (2004). Neural and psychological mechanisms underlying appetitive learning: links to drug addiction. *Curr. Opin. Neurobiol.* 14 156–162. 10.1016/j.conb.2004.03.004 15082319

[B11] Caspers S. Geyer S. Schleicher A. Mohlberg H. Amunts K. Zilles K. (2006). The human inferior parietal cortex: cytoarchitectonic parcellation and interindividual variability. *Neuroimage* 33 430–448. 10.1016/j.neuroimage.2006.06.054 16949304

[B12] Caspers S. Schleicher A. Bacha-Trams M. Palomero-Gallagher N. Amunts K. Zilles K. (2013). Organization of the human inferior parietal lobule based on receptor architectonics. *Cereb. Cortex* 23 615–628. 10.1093/cercor/bhs048 22375016 PMC3563340

[B13] Cerliani L. D’Arceuil H. de Schotten M. (2016). Connectivity-based parcellation of the macaque frontal cortex, and its relation with the cytoarchitectonic distribution described in current atlases. *Brain Struct. Funct.* 222 1331–1349. 10.1007/s00429-016-1280-3 27469273

[B14] Chikama M. McFarland N. R. Amaral D. G. Haber S. N. (1997). Insular cortical projections to functional regions of the striatum correlate with cortical cytoarchitectonic organization in the primate. *J. Neurosci.* 17 9686–9705. 10.1523/JNEUROSCI.17-24-09686.1997 9391023 PMC6573402

[B15] Cloutman L. L. Lambon Ralph M. A. (2012). Connectivity-based structural and functional parcellation of the human cortex using diffusion imaging and tractography. *Front. Neuroanat.* 6:e34 10.3389/fnana.2012.00034 PMC3429885 22952459

[B16] Cohen M. X. Schoene-Bake J. C. Elger C. E. Weber B. (2009). Connectivity-based segregation of the human striatum predicts personality characteristics. *Nat. Neurosci.* 12 32–34. 10.1038/nn.2228 19029888

[B17] Eickhoff S. B. Thirion B. Varoquaux G. Bzdok D. (2015). Connectivity-based parcellation: critique and implications. *Hum. Brain. Mapp.* 36 4771–4792. 10.1002/hbm.22933 26409749 PMC6869530

[B18] Fan L. Li H. Zhuo J. Zhang Y. Wang J. Chen L. (2016). The human brainnetome atlas: a new brain atlas based on connectional architecture. *Cereb. Cortex* 26 3508–3526. 10.1093/cercor/bhw157 27230218 PMC4961028

[B19] Ferry A. T. Ongür D. An X. Price J. L. (2000). Prefrontal cortical projections to the striatum in macaque monkeys: evidence for an organization related to prefrontal networks. *J. Comp. Neurol.* 425 447–470. 10972944 10.1002/1096-9861(20000925)425:3<447::aid-cne9>3.0.co;2-v

[B20] Floresco S. B. (2015). The nucleus accumbens: an interface between cognition, emotion, and action. *Annu. Rev. Psychol.* 66 25–52. 10.1146/annurev-psych-010213-115159 25251489

[B21] Frey S. Pandya D. N. Chakravarty M. M. Bailey L. Petrides M. Collins D. L. (2011). An MRI based average macaque monkey stereotaxic atlas and space (MNI monkey space). *Neuroimage* 55 1435–1442. 10.1146/10.1016/j.neuroimage.2011.01.040 21256229

[B22] Glickfeld L. L. Andermann M. L. Bonin V. Reid R. C. (2013). Cortico-cortical projections in mouse visual cortex are functionally target specific. *Nat. Neurosci.* 16 219–226. 10.1038/nn.3300 23292681 PMC3808876

[B23] Greve D. N. Fischl B. (2009). Accurate and robust brain image alignment using boundary-based registration. *Neuroimage* 48 63–72. 10.1016/j.neuroimage.2009.06.060 19573611 PMC2733527

[B24] Groenewegen H. J. Berendse H. W. Wolters J. G. Lohman A. H. M. (1990). The anatomical relationship of the prefrontal cortex with the striatopallidal system, the thalamus and the amygdala: evidence for a parallel organization. *Prog. Brain Res.* 85 95–118. 10.1016/S0079-6123(08)62677-1 2094917

[B25] Haber S. N. Kunishio K. Mizobuchi M. Lynd-Balta E. (1995). The orbital and medial prefrontal circuit through the primate basal ganglia. *J. Neurosci.* 15 4851–4867. 10.1523/JNEUROSCI.15-07-04851.1995 7623116 PMC6577885

[B26] Haber S. N. Lynd E. Klein C. Groenewegen H. J. (1990). Topographic organization of the ventral striatal efferent projections in the rhesus monkey: an anterograde tracing study. *J. Comp. Neurol.* 293 282–298. 10.1002/cne.902930210 19189717

[B27] Haber S. N. McFarland N. R. (1999). The concept of the ventral striatum in nonhuman primates. *Ann. N. Y. Acad. Sci.* 877 33–48. 10.1111/j.1749-6632.1999.tb09259.x 10415641

[B28] Heimer L. De Olmos J. S. Alheid G. F. Person J. Sakamoto N. Shinoda K. (1999). “The human basal forebrain. Part II,” in *Handbook of Chemical Neuroanatomy*, eds Bloom F. E. Bjorkland A. Hokfelt T. (Amsterdam: Elsevier), 57–226. 10.1016/s0924-8196(99)80024-4

[B29] Heimer L. Zahm D. S. Churchill L. Kalivas P. W. Wohltmann C. (1991). Specificity in the projection patterns of accumbal core and shell in the rat. *Neuroscience* 41 89–125. 10.1016/0306-4522(91)90202-Y 2057066

[B30] Herkenham M. Edley S. M. Stuart J. (1984). Cell clusters in the nucleus accumbens of the rat, and the mosaic relationship of opiate receptors, acetylcholinesterase and subcortical afferent terminations. *Neuroscience* 11 561–593. 10.1016/0306-4522(84)90045-9 6325999

[B31] Hilgetag C. C. Grant S. (2010). Cytoarchitectural differences are a key determinant of laminar projection origins in the visual cortex. *Neuroimage* 51 1006–1017. 10.1016/j.neuroimage.2010.03.006 20211270

[B32] Humphries M. D. Prescott T. J. (2010). The ventral basal ganglia, a selection mechanism at the crossroads of space, strategy, and reward. *Prog. Neurobiol.* 90 385–417. 10.1016/j.pneurobio.2009.11.003 19941931

[B33] Ikemoto S. (2007). Dopamine reward circuitry: two projection systems from the ventral midbrain to the nucleus accumbens-olfactory tubercle complex. *Brain Res. Rev.* 56 27–78. 10.1016/j.brainresrev.2007.05.004 17574681 PMC2134972

[B34] Ikemoto S. Qin M. Liu Z. H. (2005). The functional divide for primary reinforcement of D-amphetamine lies between the medial and lateral ventral striatum: is the division of the accumbens core, shell, and olfactory tubercle valid? *J. Neurosci.* 25 5061–5065. 10.1523/JNEUROSCI.0892-05.2005 15901788 PMC1360206

[B35] Janssen R. J. Jylanki P. Kessels R. P. C. van Gerven M. A. J. (2015). Probabilistic model-based functional parcellation reveals a robust, fine-grained subdivision of the striatum. *Neuroimage* 119 398–405. 10.1016/j.neuroimage.2015.06.084 26163800

[B36] Jay T. M. Witter M. P. (1991). Distribution of hippocampal CA1 and subicular efferents in the prefrontal cortex of the rat studied by means of anterograde transport of Phaseolus vulgaris-leucoagglutinin. *J. Comp. Neurol.* 313 574–586. 10.1002/cne.903130404 1783682

[B37] Jbabdi S. Sotiropoulos S. N. Savio A. M. Grana M. Behrens T. E. (2012). Model-based analysis of multishell diffusion MR data for tractography: how to get over fitting problems. *Magn. Reson. Med.* 68 1846–1855. 10.1002/mrm.24204 22334356 PMC3359399

[B38] Jenkinson M. Bannister P. Brady J. Smith S. (2002). Improved optimization for the robust and accurate linear registration and motion correction of brain images. *Neuroimage* 17 825–841. 10.1006/nimg.2002.1132 12377157

[B39] Johansen-Berg H. Behrens T. E. Robson M. D. Drobnjak I. Rushworth M. F. Brady J. M. (2004). Changes in connectivity profiles define functionally distinct regions in human medial frontal cortex. *Proc. Natl. Acad. Sci. U.S.A.* 101 13335–13340. 10.1073/pnas.0403743101 15340158 PMC516567

[B40] Jongen-Rêlo A. L. Voorn P. Groenewegen H. J. (1994). Immunohistochemical characterization of the shell and core territories of the nucleus accumbens in the rat. *Eur. J. Neurosci.* 6 1255–1264. 10.1111/j.1460-9568.1994.tb00315.x 7526940

[B41] Knosche T. R. Tittgemeyer M. (2011). The role of long-range connectivity for the characterization of the functional-anatomical organization of the cortex. *Front. Syst. Neurosci.* 5:58. 10.3389/fnsys.2011.00058 21779237 PMC3133730

[B42] Li H. Fan L. Zhuo J. Wang J. Zhang Y. Yang Z. (2017). ATPP: a pipeline for automatic tractography-based brain parcellation. *Front. Neuroinform.* 11:35. 10.3389/fninf.2017.00035 28611620 PMC5447055

[B43] Loonen A. J. Ivanova S. A. (2016). Circuits regulating pleasure and happiness in major depression. *Med. Hypothes.* 87 14–21. 10.1016/j.mehy.2015.12.013 26826634

[B44] Mars R. B. Sallet J. Neubert F.-X. Rushworth M. F. (2013). Connectivity profiles reveal the relationship between brain areas for social cognition in human and monkey temporoparietal cortex. *Proc. Natl. Acad. Sci. U.S.A.* 110 10806–10811. 10.1073/pnas.1302956110 23754406 PMC3696774

[B45] Mars R. B. Sallet J. Schuffelgen U. Jbabdi S. Toni I. Rushworth M. F. S. (2012). Connectivity-based subdivisions of the human right “temporoparietal junction area”: evidence for different areas participating in different cortical networks. *Cereb. Cortex* 22 1894–1903. 10.1093/cercor/bhr268 21955921

[B46] Mars R. B. Verhagen L. Gladwin T. E. Neubert F.-X. Sallet J. Rushworth M. F. (2016). Comparing brains by matching connectivity profiles. *Neurosci. Biobehav. Rev.* 60 90–97. 10.1016/j.neubiorev.2015.10.008 26627865 PMC6485474

[B47] Mavridis I. Anagnostopoulou S. (2013). Deep brain stimulation and obesity. *J. Neurosurg.* 118 485–487. 10.3171/2009.8.JNS091131 23240703

[B48] Meredith G. E. Pattiselanno A. Groenewegen H. J. Haber S. N. (1996). Shell and core in monkey and human nucleus accumbens identified with antibodies to calbindin-D28k. *J. Comp. Neurol.* 365 628–639. 8742307 10.1002/(SICI)1096-9861(19960219)365:4<628::AID-CNE9>3.0.CO;2-6

[B49] Mesulam M. M. (2006). “Foreword,” in *Fiber Pathways of the Brain*, eds Schmahmann J. D. Pandya D. N. (New York, NY: Oxford University Press), ix–x.

[B50] Moerel M. De Martino F. Formisano E. (2014). An anatomical and functional topography of human auditory cortical areas. *Front. Neurosci.* 8:225. 10.3389/fnins.2014.00225 25120426 PMC4114190

[B51] Morrison S. E. McGinty V. B. du Hoffmann J. Nicola S. M. (2017). Limbic-motor integration by neural excitations and inhibitions in the nucleus accumbens. *J. Neurophysiol.* 118 2549–2567. 10.1152/jn.00465.2017 28794196 PMC5668465

[B52] Neto L. L. Oliveira E. Correia F. Ferreira A. G. (2008). The human nucleus accumbens: where is it? A stereotactic, anatomical and magnetic resonance imaging study. *Neuromodulation* 11 13–22. 10.1111/j.1525-1403.2007.00138.x 22150987

[B53] Neubert F. Mars R. B. Sallet J. Rushworth M. F. S. (2015). Connectivity reveals relationship of brain areas for reward-guided learning and decision making in human and monkey frontal cortex. *Proc. Natl. Acad. Sci. U.S.A.* 112 2695–2704. 10.1073/pnas.1410767112 25947150 PMC4443352

[B54] Neubert F. X. Mars R. B. Thomas A. G. Sallet J. Rushworth M. F. S. (2014). Comparison of human ventral frontal cortex areas for cognitive control and language with areas in monkey frontal cortex. *Neuron* 81 700–713. 10.1016/j.neuron.2013.11.012 24485097

[B55] Nichols T. Brett M. Andersson J. Wager T. Poline J. B. (2005). Valid conjunction inference with the minimum statistic. *Neuroimage* 25 653–660. 10.1016/j.neuroimage.2004.12.005 15808966

[B56] O’Donnell P. (1999). Ensemble coding in the nucleus accumbens. *Psychobiology* 27 187–197. 10.3758/BF03332113 11797085

[B57] Passingham R. E. Stephan K. E. Kotter R. (2002). The anatomical basis of functional localization in the cortex. *Nat. Rev. Neurosci.* 3 606–616. 10.1038/nrn893 12154362

[B58] Paxinos G. Huang X.-F. Toga A. W. (2009). *The Rhesus Monkey Brain in Stereotaxic Coordinates*, 2nd Edn San Diego, CA: Academic Press.

[B59] Pennartz C. M. Groenewegen H. J. da Silva F. H. L. (1994). The nucleus accumbens as a complex of functionally distinct neuronal ensembles: an integration of behavioural, electrophysiological and anatomical data. *Prog. Neurobiol.* 42 719–761. 10.1016/0301-0082(94)90025-6 7938546

[B60] Poletti C. E. Creswell G. (1977). Fornix system efferent projections in the squirrel monkey: an experimental degeneration study. *J. Comp. Neurol.* 175 101–128. 10.1002/cne.901750107 407267

[B61] Rohlfing T. Kroenke C. D. Sullivan E. V. Dubach M. F. Bowden D. M. Grant K. A. (2012). The INIA19 template and neuromaps atlas for primate brain image parcellation and spatial normalization. *Front. Neuroinform.* 6:27. 10.3389/fninf.2012.00027 23230398 PMC3515865

[B62] Russchen F. T. Bakst I. Amaral D. G. Price J. (1985). The amygdalostriatal projections in the monkey. an anterograde tracing study. *Brain Res.* 329 241–257. 10.1016/0006-8993(85)90530-X 3978445

[B63] Salgado S. Kaplitt M. G. (2015). The nucleus accumbens: a comprehensive review. *Stereotact. Funct. Neurosurg.* 93 75–93. 10.1159/000368279 25720819

[B64] Scannell J. W. Blakemore C. Young M. P. (1995). Analysis of connectivity in the cat cerebral cortex. *J. Neurosci.* 15 1463–1483. 10.1523/JNEUROSCI.15-02-01463.1995 7869111 PMC6577853

[B65] Sjulson L. Peyrache A. Cumpelik A. Cassataro D. Buzsáki G. (2018). Cocaine place conditioning strengthens location-specific hippocampal coupling to the nucleus accumbens. *Neuron* 98 926–934. 10.1016/j.neuron.2018.04.015 29754750 PMC6154491

[B66] Stopper C. M. Floresco S. B. (2011). Contributions of the nucleus accumbens and its subregions to different aspects of risk-based decision making. *Cogn. Affect. Behav. Neurosci.* 11 97–112. 10.3758/s13415-010-0015-9 21264647

[B67] Swanson L. W. Cowan W. M. (1977). An autoradiographic study of the organization of the efferent connections of the hippocampal formation in the rat. *J. Comp. Neurol.* 172 49–84. 10.1002/cne.901720104 65364

[B68] Thiebaut de Schotten M. Croxson P. L. Mars R. B. (2018). Large-scale comparative neuroimaging: where are we and what do we need? *Cortex* [Epub ahead of print]. 10.1016/j.cortex.2018.11.028 PMC6699599 30661736

[B69] Tomassini V. Jbabdi S. Klein J. C. Behrens T. E. J. Pozzilli C. Matthews P. M. (2007). Diffusion-weighted imaging tractography-based parcellation of the human lateral premotor cortex identifies dorsal and ventral subregions with anatomical and functional specializations. *J. Neurosci.* 27 10259–10269. 10.1523/JNEUROSCI.2144-07.2007 17881532 PMC6672665

[B70] Totterdell S. Meredith G. E. (1997). Topographical organization of projections from the entorhinal cortex to the striatum of the rat. *Neuroscience.* 78 715–729. 10.1016/S0306-4522(96)00592-1 9153653

[B71] Tziortzi A. C. Haber S. N. Searle G. E. Tsoumpas C. Long C. J. Shotbolt P. (2013). Connectivity-based functional analysis of dopamine release in the striatum using diffusion-weighted MRI and positron emission tomography. *Cereb. Cortex* 24 1165–1177. 10.1093/cercor/bhs397 23283687 PMC3977617

[B72] van den Heuvel M. P. Scholtens L. H. Feldman Barrett L. Hilgetag C. C. de Reus M. A. (2015). Bridging cytoarchitectonics and connectomics in human cerebral cortex. *J. Neurosci.* 35 13943–13948. 10.1523/JNEUROSCI.2630-15.2015 26468195 PMC6608182

[B73] van der Plasse G. Schrama R. van Seters S. P. Vanderschuren L. J. M. J. Westenberg H. G. M. (2012). Deep brain stimulation reveals a dissociation of consummatory and motivated behavior in the medial and lateral nucleus accumbens shell of the rat. *PLoS. One* 7:33455. 10.1371/journal.pone.0033455 22428054 PMC3302768

[B74] van Dongen Y. C. Deniau J. M. Pennartz C. M. Galis-de G. Y. Voorn P. Thierry A. M. (2005). Anatomical evidence for direct connections between the shell and core subregions of the rat nucleus accumbens. *Neuroscience* 136 1049–1071. 10.1016/j.neuroscience.2005.08.050 16226842

[B75] Van Essen D. C. Smith S. M. Barch D. M. Behrens T. E. Yacoub E. Ugurbil K. (2013). The WU-Minn human connectome project: an overview. *Neuroimage* 80 62–79. 10.1016/j.neuroimage.2013.05.041 23684880 PMC3724347

[B76] van Groen T. Wyss J. M. (1990). Extrinsic projections from area CA1 of the rat hippocampus: olfactory, cortical, subcortical, and bilateral hippocampal formation projections. *J. Comp. Neurol.* 302 515–528. 10.1002/cne.903020308 1702115

[B77] Van Hoesen G. W. Yeterian E. H. Lavizzo-Mourey R. (1981). Widespread corticostriate projections from temporal cortex of the rhesus monkey. *J. Comp. Neurol.* 199 205–219. 10.1002/cne.901990205 7251940

[B78] Voorn P. Brady L. S. Berendse H. W. Richfield E. K. (1996). Densitometrical analysis of opioid receptor ligand binding in the human striatum - I. Distribution of u opioid receptor defines shell and core of the ventral striatum. *Neuroscience* 75 777–792. 10.1016/0306-4522(96)00271-0 8951872

[B79] Wang J. Fan L. Wang Y. Xu W. Jiang T. Fox P. T. (2015). Determination of the posterior boundary of Wernicke’s area based on multimodal connectivity profiles. *Hum. Brain. Mapp.* 36 1908–1924. 10.1002/hbm.22745 25619891 PMC4782781

[B80] Wang J. Fan L. Zhang Y. Liu Y. Jiang D. Zhang Y. (2012). Tractography-based parcellation of the human left inferior parietal lobule. *Neuroimage* 63 641–652. 10.1016/j.neuroimage.2012.07.045 22846658

[B81] Wang J. Zuo Z. Xie S. Miao Y. Ma Y. Zhao X. (2017). Parcellation of macaque cortex with anatomical connectivity profiles. *Brain Topog.* 31 161–173. 10.1007/s10548-017-0576-9 28707157

[B82] Xia X. Fan L. Cheng C. Eickhoff S. B. Chen J. Li H. (2017). Multimodal connectivity-based parcellation reveals a shell-core dichotomy of the human nucleus accumbens. *Hum. Brain Mapp.* 38 3878–3898. 10.1002/hbm.23636 28548226 PMC5685173

[B83] Yin H. H. Knowlton B. J. (2006). The role of the basal ganglia in habit formation. *Nat. Rev. Neurosci.* 7 464–476. 10.1038/nrn1919 16715055

[B84] Zahm D. S. (1999). Functional-anatomical implications of the nucleus accumbens core and shell subterritories. *Ann. N. Y. Acad. Sci.* 877 113–128. 10.1111/j.1749-6632.1999.tb09264.x 10415646

[B85] Zhang Y. Brady M. Smith S. (2001). Segmentation of brain MR images through a hidden Markov random field model and the expectation-maximization algorithm. *IEEE. Trans. Med. Imaging* 20 45–57. 10.1109/42.906424 11293691

[B86] Zhao X. Yang R. Wang K. Zhang Z. Wang J. Tan X. (2018). Connectivity-based parcellation of the nucleus accumbens into core and shell portions for stereotactic target localization and alterations in each NAc subdivision in mTLE patients. *Hum. Brain Mapp.* 39 1232–1245. 10.1002/hbm.23912 29266652 PMC6866435

[B87] Zhou L. Furuta T. Kaneko T. (2003). Chemical organization of projection neurons in the rat accumbens nucleus and olfactory tubercle. *Neuroscience* 120 783–798. 10.1016/S0306-4522(03)00326-9 12895518

[B88] Zhuo J. Fan L. Liu Y. Zhang Y. Yu C. Jiang T. (2016). Connectivity profiles reveal a transition subarea in the parahippocampal region that integrates the anterior temporal-posterior medial systems. *J. Neurosci.* 36 2782–2795. 10.1523/JNEUROSCI.1975-15.2016 26937015 PMC6604873

[B89] Zuo X. Di Martino A. Kelly C. Shehzad Z. E. Gee D. G. Klein D. F. (2010). The oscillating brain: complex and reliable. *Neuroimage* 49 1432–1445. 10.1016/j.neuroimage.2009.09.037 19782143 PMC2856476

