# Supplementary material for: Fine-Grained Parcellation of the Macaque Nucleus Accumbens by High-Resolution Diffusion Tensor Tractography
Source: Front Neurosci. 2019 Jul 10;13:709. doi: 10.3389/fnins.2019.00709 (PMC6635473; doi:10.3389/fnins.2019.00709)
Supplement: Supplementary file 1 [file Data_Sheet_1.docx]

Supplementary Material

# Boundaries of the Acb Suggested by Anatomical Analyses

As summarized by Salgado and Kaplitt (2015), the boundaries of the anatomical Acb region suggested by more modern anatomical analyses could be described as follows: (1) posterior limit: the posterior border of the anterior commissure (Heimer, 2000; Neto et al., 2008); (2) anterior limit: where the rostral limit of the internal capsule starts separating the caudate from the putamen (Neto et al., 2008); (3) medial limit: the sagittal plane passing by the inferior border of the lateral ventricle; (4) lateral limit: a line extending downwards and laterally to the rostral edge of the internal capsule; (5) dorsal limit: the horizontal plane passing under the caudate nucleus head from the inferior border of the lateral ventricle to the inferior limit of the internal capsule, and (6) ventral limit: the external capsule (lateral side) and Broca’s diagonal band (medial side) anteriorly, the anterior hypothalamic nucleus posteriorly (Neto et al., 2008).

# Preparation of the Specimens and MRI Data Acquisition

High-resolution adult rhesus macaque (Table S1) MRI dataset1 (MDS1) consisted of 8 brain specimens. These monkeys were obtained from a colony maintained by Kunming Institute of Zoology, Chinese Academy of Sciences, and were judged by the veterinarian as appropriate subjects for euthanasia due to serious physical diseases (acute gastroenteritis and enteritis). The macaques were administered an overdose of pentobarbital (100 mg/kg). After verifying the status of deep anesthesia, they were transcardially perfused with phosphate-buffered saline (PBS) solution containing 1% heparin (PH7.4), followed by a pre-cooled PBS solution containing 4% paraformaldehyde. 5 minutes after beginning the perfusion, the speed was lowered to 1 ml/min, which was maintained for 2 hours. The heads were then removed from the bodies and stored in PBS solution containing 4% paraformaldehyde. The skull was carefully removed to expose the whole brain and was transferred to an MRI compatible holder, bundled with medical gauze, and immersed in Fomblin (Solvay, Brussels, Belgium) to prevent dehydration and susceptibility to artifacts. No apparent structural anomaly was found in any of the brains used in the present study.

**Table S1.** Descriptive information for the 8 rhesus macaque brain specimens

| **Perfusion date (year-month-day)** | **No.** | **Sex** | **Age (years)** | **Body weight (kg)** |
| --- | --- | --- | --- | --- |
| 2016-05-09 | 93310 | female | 23 | 3.24 |
|  | 08046 | female | 8 | 3.58 |
|  | 12027 | male | 4 | 3.06 |
|  | 12411 | male | 4 | 2.89 |
| 2016-05-10 | 01006 | female | 15 | 3.57 |
|  | 04084 | female | 12 | 4.23 |
|  | 10427 | female | 6 | 3.69 |
|  | 11402 | female | 5 | 2.90 |

* Note: Their body weights are significantly lower than the normal rhesus macaque due to diseases.

# MRI Data Acquisition

MRI data were performed on a 9.4T horizontal animal MRI system (Bruker Biospec 94/30 USR) with Paravision 6.0.1, the gradients are equipped with slew rate of 1170 mT/m/ms and maximum strength 300 mT/m, radiofrequency transmission and reception were achieved with a 154 mm inner-diameter quadrature radiofrequency coil. T2w images were acquired using 2D Turbo RARE sequence with TR/TE = 8464/30.9 ms, flip angle = 90°, matrix = 280×220, FOV = 84×66 mm, slice thickness = 0.6mm, number of excitations = 4. DTI images were acquired using a 2D diffusion weighted spin echo pulse sequence, TR/TE = 9800/21.8 ms, bandwidth = 304 kHz, FOV = 94×66 mm, matrix = 140×110, and voxel sizes = 0.6×0.6×0.6577 mm. 60 diffusion directions with b = 1000 s/mm^2^ (Δ/δ = 10.9/4.5 ms, maximum gradient amplitude = 276 mT/m) and 4 non-diffusion gradients (b = 0 s/mm^2^) acquisition, and time cost was approximately 115 hours per specimen. The data quality of these diffusion images were analyzed in Section 4.

# MRI Data Quality Checking

The visual inspection was first performed for these MRI data to ensure there were no obvious artefacts and geometric distortions caused by living bodies’ motion and physiological noise, as well as the static magnetic field inhomogeneity and the eddy current, etc.

For the ex vivo macaque diffusion MRI data, the *b*-value was not set to the 4000 s/mm^2^ recommended in previous studies (D’ Arceuil et al., 2007; Dyrby et al., 2011) but at the relatively low value of 1000 s/mm^2^ like some other studies (see Table S2). For example, Calabrese et al. (2015) reconstructed 42 major white matter tracks and even several smaller pathways (e.g., the cranial nerves, the fasciculus retroflexus, and the stria medullaris) using diffusion images with the relatively low *b*-value of 1500 s/mm^2^. In this study, a preliminary test of the data quality was performed for these diffusion images using a typical signal-to-noise ratio (SNR). Further testing was performed by comparing the parcellation results generated using different *b*-values (1000, 2000, 3000, and 4000 s/mm^2^) for the diffusion images.

**Table S2.** Previous studies using relatively low *b*-value for tractographic reconstruction

| **Authors** | **Scanner** | **Subjects** | **DTI Sequence** | **TR/TE (ms)** | **# Dir.** | **Max *b*-value (s/mm^2^) & other parameters** |
| --- | --- | --- | --- | --- | --- | --- |
| Calabrese et al., 2016 | Agilent 7T field strength 650 mT/m gradient 65mm QUA. Coil | 10 rhesus monkey brain specimens | 3D diffusion weighted spin echo | 100/21.5 | 12 | **1500** Δ = 14 ms δ = 4 ms amplitude = 50 G/cm |
| Rane et al., 2010 | Siemens TRIO 3T | 4 rhesus monkey brain specimens | STEAM EPI; STEAM EPI; double spin-echo | 1500/80; 1500/80; 1500/104 | 30; 30; 30 | **1700**, δ = 21 ms, diffusion time = 48 ms; **1700**, δ = 21 ms, diffusion time = 192 ms; **1700**, δ = 21 ms, effective diffusion time = 45 ms |
| Budde et al., 2011 | Bruker Biospec 7T field strength;  microimaging gradient insert and a 20-mm probe. | 5 (8-12 week-old; female) Wistar rats brain specimens | A multiple-echo pulsed gradient spin echo sequence | 4000/20 | 30 | **1200** Δ = 10 ms δ = 4 ms |
| Feng et al., 2017 | A Bruker 4.7 T scanner | 10 (age: 5.3 ± 2.8 years; body  weight = 5.67 ± 2.34 kg; 6 male) macaques | 3D multiple spin echo diffusion tensor sequence | 700/32.5 | 8 | **1000** |

The diffusion tensor is reconstructed by evaluating the signal loss in diffusion weighted images with respect to the signal of a reference image (b = 0 s/mm^2^) with no diffusion attenuation. Hence, the low difference signal can be overwhelmed by noise, which adds to that of the diffusion weighted image and the reference one. The insufficient SNR can present a critical problem for the subsequent tractographic reconstruction and eventual parcellation. The SNRs of the macaque diffusion images were calculated and checked using a single image-based two-region method (Dietrich et al., 2007; Griffanti et al., 2012). Specifically, for each macaque, two separate tissues, the gray matter (GM) and white matter (WM), were extracted in subject-native diffusion space to estimate the signals; a region positioned in the background was used to evaluate the noise. The SNRs were then calculated for these diffusion images (Fig. S1). As a rule of thumb, the SNR of the diffusion MRI acquisition image should be at least 20 to derive relatively unbiased measures of parameters, such as the fractional anisotropy (Mukherjee et al., 2008). All the diffusion images in this study met this requirement and thus had an appropriate SNR for the subsequent tractographic reconstruction.


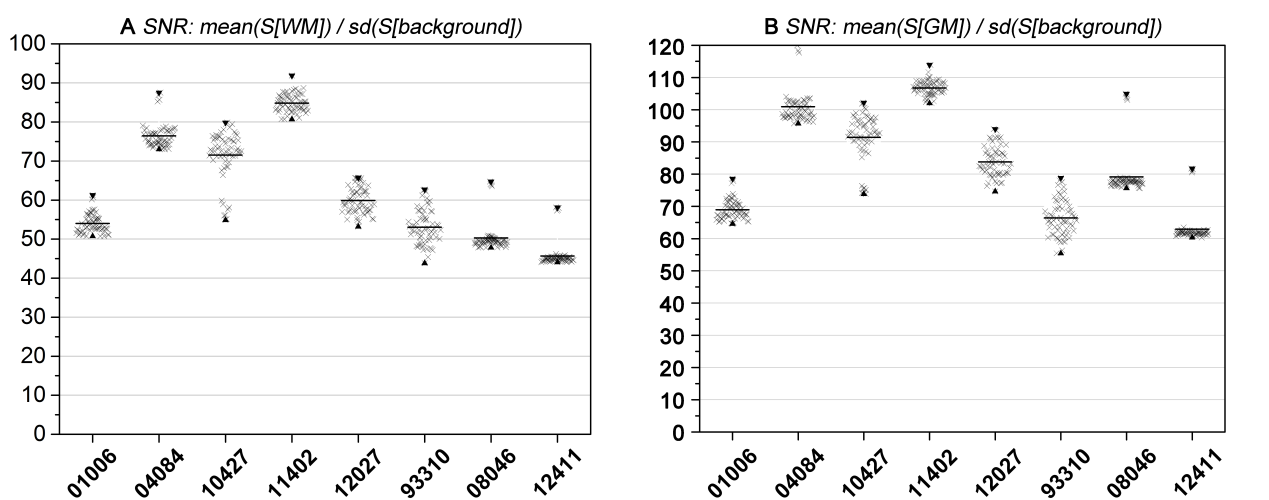


**Figure S1.** Scatter plot visualizing the SNRs generated by the WM (A) and the GM (B). The horizontal axis indicates 8 macaque brain specimens. ‘X’ marks showing the SNR value of the diffusion images (64 values per subject). The mean and extreme (maximum and minimum) SNRs are indicated by solid line and triangular symbols, respectively. All these SNRs are far above 20.

The primary purpose of this study was the tractography-based parcellation. Therefore, we performed a further test of the data availability by comparing the similarities between the parcellation results generated by different *b*-values. For this purpose, we prepared another macaque brain specimen using the above-mentioned operations and used it to acquire four sets of diffusion MRI data at *b*-values of 1000, 2000, 3000, and 4000 s/mm^2^ while keeping all the other parameters unchanged. Then, the nucleus accumbens (Acb) was chosen as seed to construct the tractography-based parcellation using these four sets of data (the individual parcellation procedure was detailed in main text). The parcellation results (Fig. S2) had similar subregions distribution pattern across the MRI data for the different *b*-values (1000, 2000, 3000, and 4000 s/mm^2^), at least in 2-, 3-, 4-, 5-, and 6-cluster solutions. In particular, the 2-, 3-, and 4-cluster solutions results from the low *b*-value (e.g., 1000 s/mm^2^) diffusion MRI data seemed to present a high degree of overlap with those from the higher *b*-values (e.g., 4000 s/mm^2^) diffusion data, suggesting that low *b*-value diffusion data is feasible for tractography-based parcellations.

Taking above quality tests into account, the macaque diffusion MRI data acquired in this study at a relative low *b*-value of 1000 s/mm^2^ had an appropriate SNR for tractographic reconstruction and could also enable us to obtain parcellation results that are consistent with those that would have been obtained using higher *b*-values data. Thus, we conclude that the other 8 *ex vivo* macaque diffusion data should have the same ability to support the parcellation research.

**
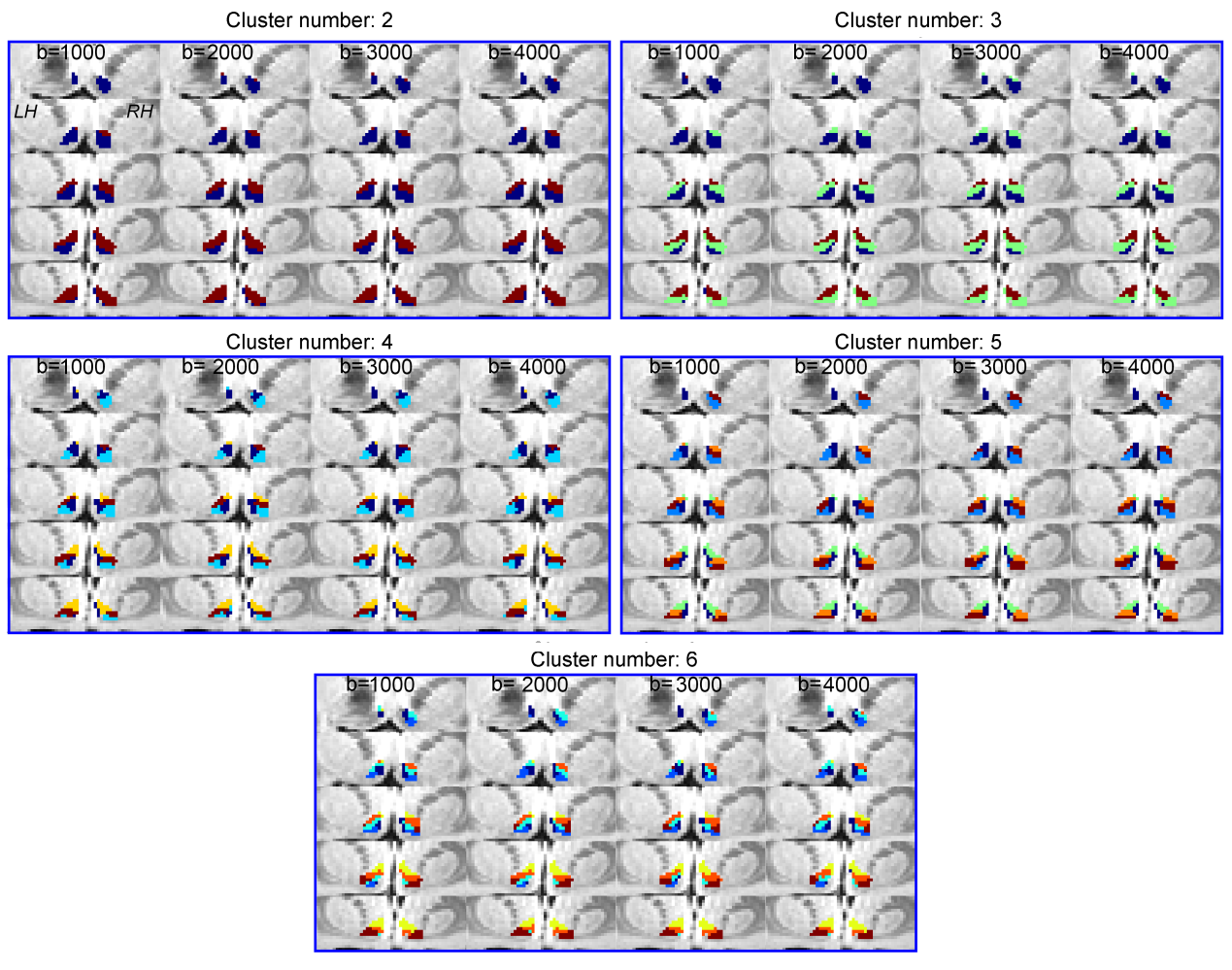
Figure S2.** The parcellation results of the Acb. Different diffusion MRI data (*b*-values = 1000, 2000, 3000, and 4000 s/mm^2^) were used for this procedure and the results are shown here for comparison. The Acb was parcellated into 2, 3, 4, 5, and 6 clusters. The parcellation results show similar Acb subregions spatial distribution patterns across datasets. Left hemisphere, LH; right hemisphere, RH.

# Necessary Measures to Parcellate the Acb

The down-sample of the tractographic images of voxels will inevitably cause a loss of connectivity details, and thus may impact on the connectivity-based parcellation result. We added an additional test to investigate the degree of parcellating precision affected by the down-sample of the tractographic images. We took an individual Acb region as an example and parcellated this region using the whole-brain connectivity matrix formed by the down-sampled tractographic images (voxel sizes: 0.6×0.6×0.6577 mm) and the whole-brain connectivity matrix formed by the tractographic images have not yet been down-sampled (voxel sizes: 1.2×1.2×1.3154 mm) respectively. We found their well consistent parcellation results at a relative coarse-grained resolution (see the 2-cluster solution shown in Fig. S3) and similar but not identical parcellation results at a relative fine-grained resolution (see the 4-cluster solution shown in Fig. S3). In view of this, we used the down-sampled tractographic images to parcellate the striatum to define the Acb (at a coarse-grained resolution), while used the high-precision original tractographic images to investigate a finer Acb parcellation than the traditional dichotomization.


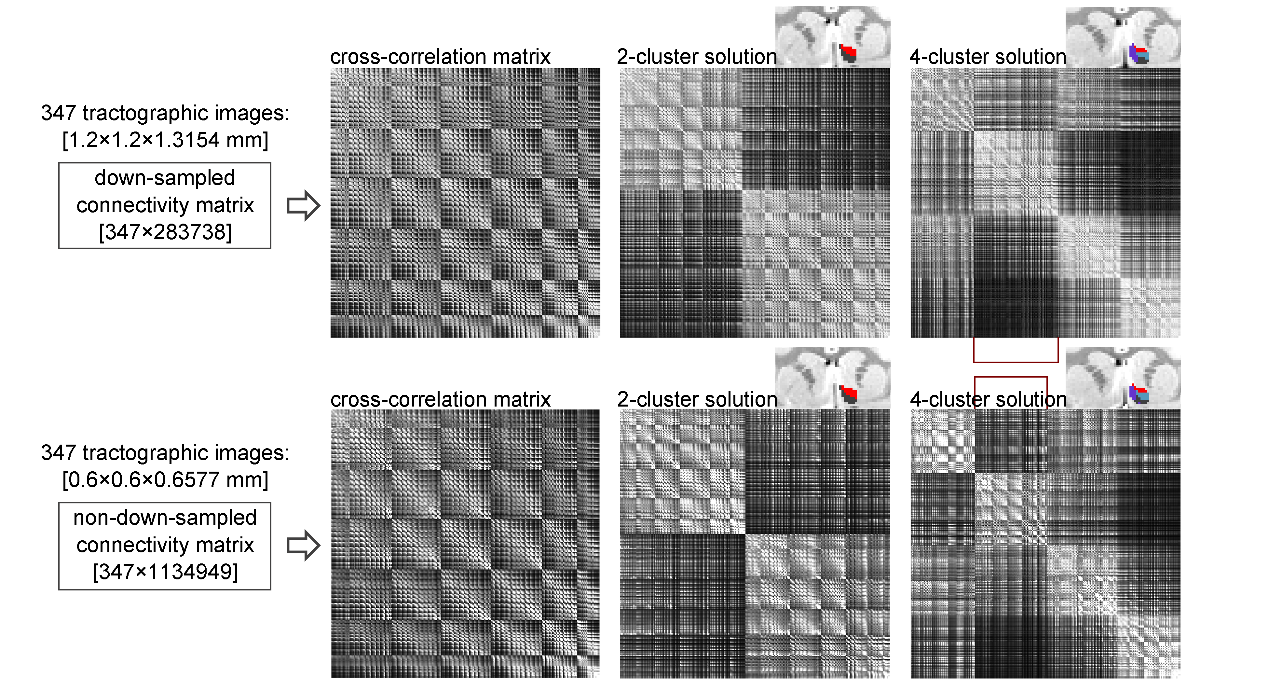


**Figure S3.** The effect of the down-sampled tractographic images on the parcellation. The original tractographic images and the down-sampled tractographic images were used to parcellate the individual (No. 11402) Acb region (347 voxels) and their results are shown in top and low panel respectively. We found similar topological distribution of the final parcellation results in the 4-cluster solution, but a few individual clusters present certain volumetric differences, i.e., different side lengths of the corresponding squares shown in right two panels.

# The ventral striatal subregions


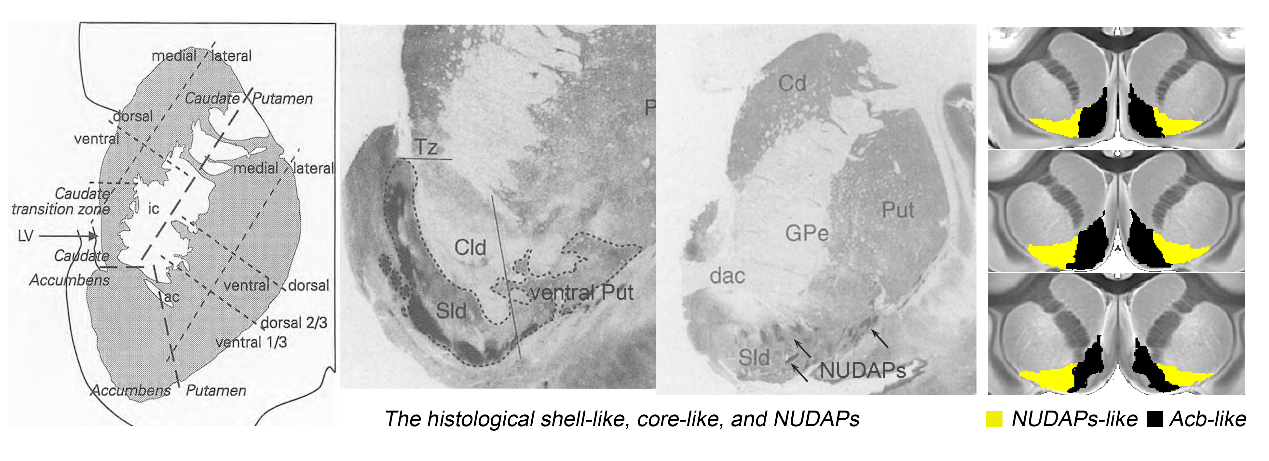


**Figure S4.** The ventral striatal subregions. The neurochemically unique domains of the accumbens and putamen (NUDAPs) in primate striatum was described as a histological region comprising of ventral one-third of the putamen and a small region of the lateral accumbens shell (Voorn et al., 1996). The counterpart region in this study was thus named NUDAPs-like division (right panel). Sld, shell-like division; Cld, core-like division; Tz, transion zone; Put, putamen.

# Definition of the criteria for the target areas

Referring to earlier literature (Cauda et al., 2011; Xia et al., 2017), we chose a set of brain areas which had ‘important’ connections with most of the two Acb subregions as target group. The ‘important’ anatomical connectivity was defined according to the following criteria: 1) The number of activation voxels included in the area surpassed a fixed fraction, > 2%, of the total voxels of that area, or 2) the number of activation voxels included in the area surpassed a fixed fraction, > 2%, of the total number of activation voxels. And the ‘important’ functional connection was defined according to the following criteria: 1) The number of activation voxels included in the area surpassed a fixed fraction, > 5%, of the total voxels of that area, or 2) the number of activation voxels included in the area surpassed a fixed fraction, > 5%, of the total number of activation voxels.


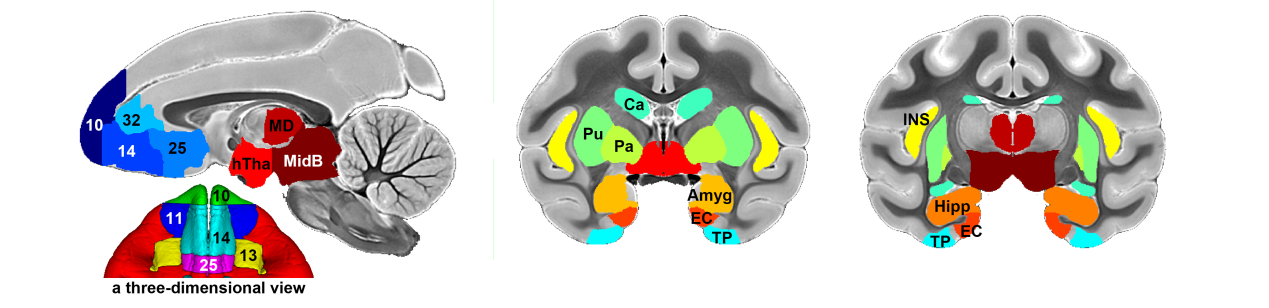


**Figure S5.** The targets family. 17 targets meeting above mentioned connection criteria were extracted from the histological atlas to construct the fingerprint framework. Acronyms: TP, temporal pole; Pa, pallidum; Ca, caudate nucleus; Pu, putamen; INS, insula cortex; Amyg, amygdala; Hipp, hippocampus; EC, entorhinal cortex; hTha, hypothalamus; MD, mediodorsal part of the thalamus; MidB, midbrain.

# The Whole-Brain Voxel-Wise of the Acb Subregions


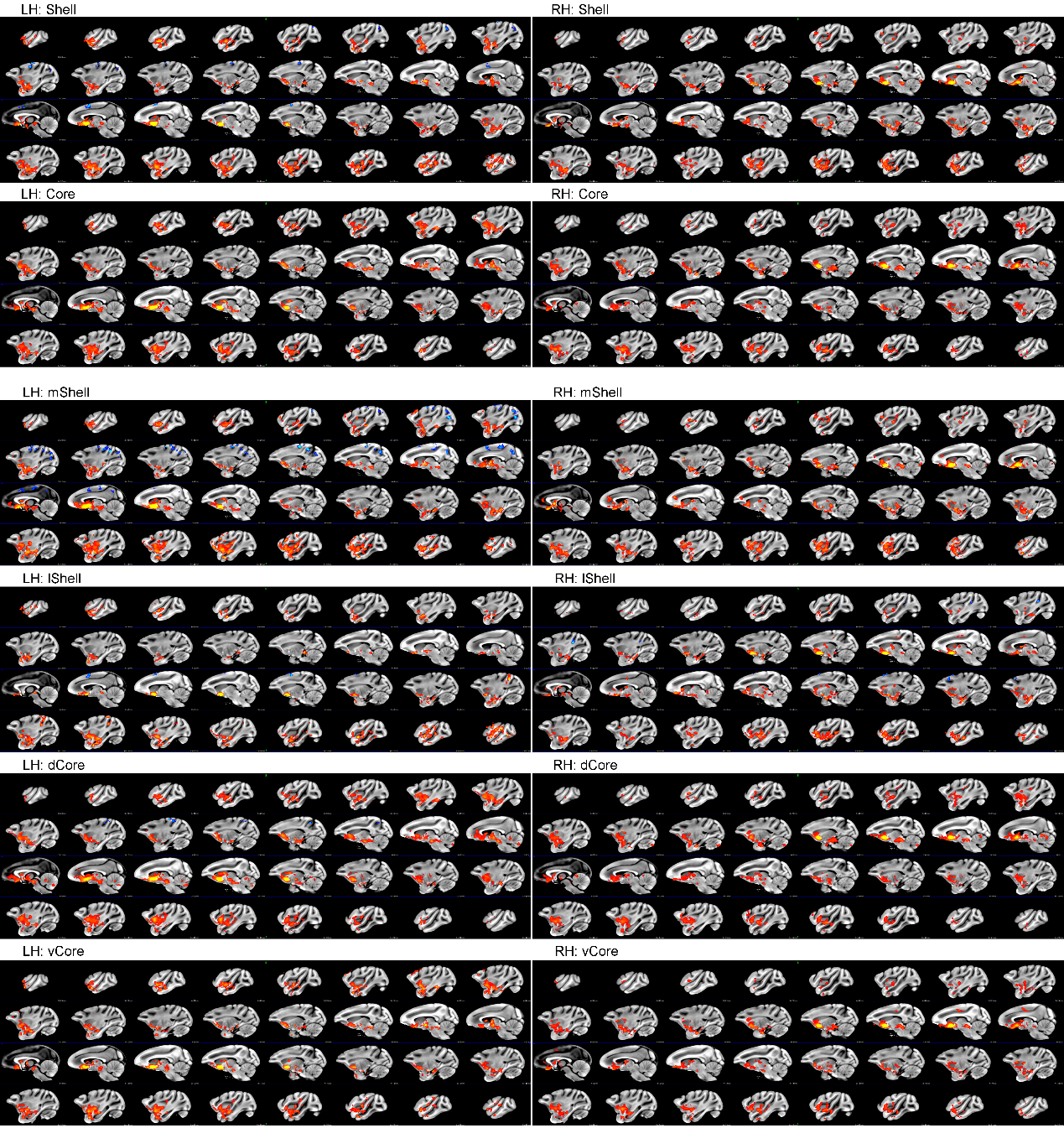


**Figure S6.** The whole-brain voxel-wise significant rsFC maps of the Acb subregions. For each Acb subregion, all individual normalized *z*-valued rsFC maps were fed into a random effects one-sample *t*-test to determine the regions that had significant correlations with this Acb subregions in a voxel-wise manner. A statistical threshold of p (uncorrected) < 0.05 was set to achieve a corrected cluster wise statistical significance of p < 0.05, with the cluster size estimated based on the group-averaged GM mask and the group-averaged Gaussian filter width.

# The RsFC between the Acb Subregions

For each pair of these Acb subregions, we extracted their mean time series using the non-smoothing fMRI data and calculated their Pearson correlation (i.e., rsFC) in each individual for subsequent statistical analyses. A contrasts rsFC was calculated using a paired samples *t*-test to compare the differences in the functional connection between all the pairs of the subregions (see Fig. S7). For each Acb subregion in the left and right hemisphere, we calculated the Pearson correlation between their mean time series in each individual and showed the statistical results in Fig. S8.


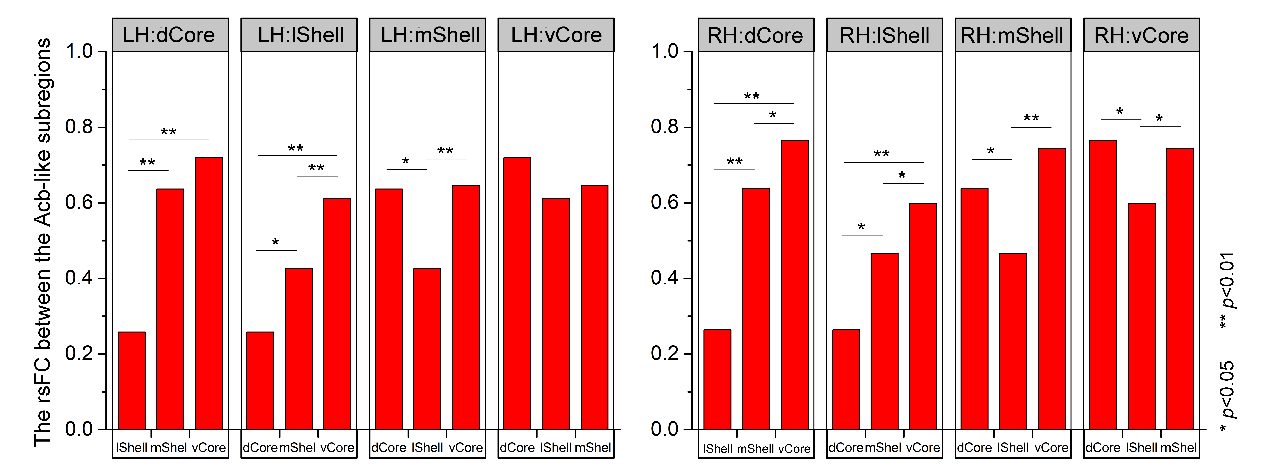


**Figure S7.** The rsFC between the four Acb-like subregions. The left and right panel indicates the rsFC in left and right hemisphere, respectively. For example, the group-level averaged rsFC between the dCore and the other three subregions in left hemisphere are shown in most left three bars. And the rsFC between the dCore and lShell is significant lower than the rsFC between the dCore and mShell.


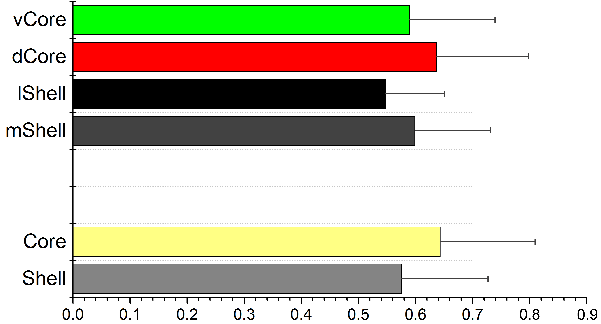


**Figure S8.** The rsFC between the corresponding Acb subregions.

# References

Budde, M. D., Janes, L., Gold, E., Turtzo, L. C., and Frank, J. A. (2011). The contribution of gliosis to diffusion tensor anisotropy and tractography following traumatic brain injury: validation in the rat using Fourier analysis of stained tissue sections. *Brain*. 134, 2248-2260. [doi: 10.1093/brain/awr161](http://doi.org/10.1093/brain/awr161)

Calabrese, E., Badea, A., Coe, C. L., Lubach, G. R., Shi, Y., Styner, M. A., et al. (2015). A diffusion tensor MRI atlas of the postmortem rhesus macaque brain. *Neuroimage*. 117, 408-416. [doi: 10.1016/j.neuroimage.2015.05.072](https://doi.org/10.1016/j.neuroimage.2015.05.072)

Cauda, F., Cavanna, A. E., D’Agata, F., Sacco, K., Duca, S., and Geminiani, G. C. (2011). Functional connectivity and coactivation of the nucleus accumbens: A combined functional connectivity and structure-based meta-analysis. *J. Cogn. Neurosci*. 23, 2864-2877. [doi: 10.1162/jocn.2011.21624](http://doi.org/10.1162/jocn.2011.21624)

D’Arceuil, H. E., Westmoreland, S., and de Crespigny, A. J. (2007). An approach to high resolution diffusion tensor imaging in fixed primate brain. *Neuroimage*. 35, 553-565. [doi: 10.1016/j.neuroimage.2006.12.028](http://doi.org/10.1016/j.neuroimage.2006.12.028)

Dietrich, O., Raya, J. G., Reeder, S. B., Reiser, M. F., and Schoenberg, S. O. (2007). Measurement of signal-to-noise ratios in MR images: influence of multichannel coils, parallel imaging, and reconstruction filters. *J. Magn. Reson. Imaging*. 26, 375-385. [doi: 10.1002/jmri.20969](http://doi.org/10.1002/jmri.20969)

Dyrby, T. B., Baare, W. F. C., Alexander, D. C., Jelsing, J., Garde, E., and Søgaard, L. V. (2011). An Ex Vivo Imaging Pipeline for Producing High-Quality and High-Resolution Diffusion-Weighted Imaging Datasets. *Hum. Brain. Mapp*. 32, 544-563. [doi: 10.1002/hbm.21043](http://doi.org/10.1002/hbm.21043)

Feng, L., Jeon, T., Yu, Q., Ouyang, M., Peng, Q., Mishra, V., et al. (2017). Population-averaged macaque brain atlas with high-resolution *ex vivo* DTI integrated into in vivo space. *Brain. Struct. Funct*. 222, 4131-4147. [doi: 10.1007/s00429-017-1463-6](http://doi.org/10.1007/s00429-017-1463-6)

Heimer, L. (2000). Basal forebrain in the context of schizophrenia. *Brain. Res. Brain. Res. Rev*. 31, 205-235. [doi: 10.1016/S0165-0173(99)00039-9](https://doi.org/10.1016/S0165-0173(99)00039-9)

Griffanti, L., Baglio, F., Preti, M. G., Cecconi, P., Rovaris, M., Baselli, G., et al. (2012). Signal-to-noise ratio of diffusion weighted magnetic resonance imaging: Estimation methods and in vivo application to spinal cord. *Biomed. Signal. Process. Control*. 7, 285-294. [doi: 10.1016/j.bspc.2011.06.003](https://doi.org/10.1016/j.bspc.2011.06.003)

Mukherjee, P., Chung, S. W., Berman, J. I., Hess, C. P., and Henry, R. G. (2008). Diffusion Tensor MR Imaging and Fiber Tractography: Technical Considerations. *AJNR. Am. J. Neuroradiol*. 29, 843-852. [doi: 10.3174/ajnr.A1052](http://doi.org/10.3174/ajnr.A1052)

Neto, L. L., Oliveira, E., Correia, F., and Ferreira, A. G. (2008). The human nucleus accumbens: where is it? A stereotactic, anatomical and magnetic resonance imaging study. *Neuromodulation*. 11, 13-22. [doi: 10.1111/j.1525-1403.2007.00138.x](http://doi.org/10.1111/j.1525-1403.2007.00138.x)

Rane, S., Nair, G., and Duong, T. Q. (2010). DTI at long diffusion time improves fiber tracking. *NMR. Biomed*. 23, 459-465. [doi: 10.1002/nbm.1482](http://doi.org/10.1002/nbm.1482)

Salgado, S., and Kaplitt, M. G. (2015). The nucleus accumbens: A comprehensive review. *Stereotact. Funct. Neurosurg*. 93, 75-93. [doi: 10.1159/000368279](http://doi.org/10.1159/000368279)

Voorn, P., Brady, L. S., Berendse, H. W., and Richfield, E. K. (1996). Densitometrical analysis of opioid receptor ligand binding in the human striatum - I. Distribution of u opioid receptor defines shell and core of the ventral striatum. *Neuroscience*. 75, 777-792. [doi: 10.1016/0306-4522(96)00271-0](https://doi.org/10.1016/0306-4522(96)00271-0)

Xia, X., Fan, L., Cheng, C., Eickhoff, S. B., Chen, J., Li, H., et al. (2017). Multimodal Connectivity-Based Parcellation Reveals a Shell-Core Dichotomy of the Human Nucleus Accumbens. *Hum. Brain. Mapp*. 38, 3878-3898. [doi: 10.1002/hbm.23636](http://doi.org/10.1002/hbm.23636)
